# Supplementary material for: Providers’ experiences with abortion care: A scoping review
Source: PLoS One. 2024 Jul 1;19(7):e0303601. doi: 10.1371/journal.pone.0303601 (PMC11216598; doi:10.1371/journal.pone.0303601)
Supplement: S2 Table — (DOCX) [file pone.0303601.s002.docx]

**S2 Table**

**Overview of the studies included in the scoping review on providers’ experiences with abortion care**

| **S2 Table.** Summary of characteristics of studies included in the scoping review. | | | | | | | | |  |
| --- | --- | --- | --- | --- | --- | --- | --- | --- | --- |
| **Ref** | **Author, Year** | **Country**  **Sample Size**  **Professions** | **Procedures Provided ^a^** | | **Methodology**  **Data Collection Method**  **Analysis** | | **Key Findings** | | **MMAT Score ^b^** |
| [1] | Chowdhary, 2022 | United States  12 providers  Physicians | Comprehensive abortion service | | Qualitative  Interviews  Grounded theory | | - Physician-identified challenges to provider recruitment and retention in the Southern US included restrictive legislation, the separation of abortion from other patient care, lack of training opportunities, safety concerns, identity struggles, and marginalisation within the medical community. - Providers experienced stigma related to their abortion work. - Motivations to continue providing despite these challenges included the high need for care, a desire to combat health access disparities, and having personal ties to the South. - They believed that regional networking and training opportunities could help. | | 4 |
| [2] | Mainey, 2022 | Australia  18 providers  Nurses, Midwives | Comprehensive abortion service | | Qualitative  Interviews  Grounded theory | | - Nurses and midwives either worked with or against their healthcare system when providing care to women affected by gender-based violence. - Where the system supported women, few issues were raised. - Where the system didn’t, providers bent or broke the law, local policy, and cultural norms to facilitate holistic care. They resolved to continue supporting women who were being failed by the system. | | 5 |
| [3] | Newton-Levinson, 2022 | United States  15 providers  Leadership, Clinic Support Staff | Comprehensive abortion service | | Qualitative  Interviews  Thematic analysis | | - High levels of staff turnover negatively impacted clinic functioning and patient care. Challenges to recruitment and retention included living the conservative south, a perceived lack of value for staff among managers, gaps in communication between providers, and living in rural area. - Proposed strategies to include recruitment and retention included investment in management, career advancement opportunities, prioritizing staff retention, and creating a space for self-care | | 4 |
| [4] | Reeves, 2022 | United States  10 providers  Anaesthetists | Comprehensive abortion service | | Qualitative  Interviews  Grounded theory | | - Anaesthetists were willing to be involved in an abortion procedure in at least some circumstances. Most were comfortable with cases of maternal or fetal health indications. - Support for abortions for social or financial reasons waned. | | 2 |
| [5] | Armour, 2021 | New Zealand  8 providers  Midwives | Second and Third-trimester abortion | | Qualitative  Interviews  Hermeneutic-phenomenological thematic analysis | | - Midwives believed that abortion care is a different role within midwifery as they encounter death instead of birth. - They said it was important to immerse themselves in the patients’ emotional space to create meaningful connections and support them. - Despite this, they felt unprepared for the emotional realities of abortion care, especially when they care for many women. | | 5 |
| **Ref** | **Author, Year** | **Country**  **Sample Size**  **Professions** | **Procedures Provided ^a^** | **Methodology**  **Data Collection Method**  **Analysis** | | **Key Findings** | | **MMAT Score ^b^** | |
| [6] | Becker, 2021 | United States  25 providers  Nurses, Patient Advocates, Recovery Room, Doula, Patient Services Director, Manager, Physician, director | Comprehensive abortion service | Qualitative  Interviews  Grounded theory | | - Providers experienced tension in the language around abortion and did not support common pro-choice rhetoric that the fetus is just a “clump of cells.” - Many believed it important to acknowledge that the potential life of the fetus. - Contact with the fetus became more challenging with increased gestation as it becomes more noticeably human, and force some to question their support. - Seeing the fetal remains is more challenging than other aspects of care that expose providers to the fetus, such as ultrasound scanning. | | 4 | |
| [7] | Cannon, 2021 | United States  39 providers  Physicians (Obstetricians, Family Medicine, other) | Comprehensive abortion service | Qualitative  Interviews  Initial coding methods | | - The physicians believed it was important to take a patient-centred approach and educating patients as highly important during contraceptive counselling. - They also believed it was their responsibility to prevent recurrent abortions. This was motivated by a despite to prevent women facing additional challenges and by discomfort about repeat abortions. | | 3 | |
| [8] | Dempsey, 2021 | Ireland  156 providers  General practitioners, Obstetricians, Midwives, Nurses, Administrators, Anesthetist | Comprehensive abortion service | Quantitative (Cross-sectional observational)  Survey  Statistical analyses | | - Providers in the Republic of Ireland experienced stigma related to their involvement in abortion care. - Levels of stigma were higher among those working in Irish hospitals, e.g. obstetricians, midwives, and nurses, compared to providers working in general practice. | | 4 | |
| [9] | Ewnetu, 2021 | Ethiopia  30 providers  Nurses, Midwives, Obstetricians, GPs, Public health specialists | Comprehensive abortion service | Qualitative  Interviews  Systematic text condensation framework | | - The law is ambiguous in its wording, with providers having space to interpret it with their professional discretion. This lack of clarity was an ethical dilemma for many providers who were unsure of who can legally access care. - All believed that abortion is justified if a diagnosis of a fetal abnormality has been made. | | 5 | |

| **Ref** | **Author, Year** | **Country**  **Sample Size**  **Professions** | **Procedures Provided ^a^** | | **Methodology**  **Data Collection Method**  **Analysis** | | **Key Findings** | | **MMAT Score ^b^** |
| --- | --- | --- | --- | --- | --- | --- | --- | --- | --- |
| [10] | Magelssen, 2021 | Ethiopia  30 providers  Nurses, Midwives, Obstetricians, GPs, Public health specialists | Comprehensive abortion service | | Qualitative  Interviews  Systematic text condensation framework | | - Providers said that conscientious objection is practiced in Ethiopia despite the law forbidding its practice. - Those in hospitals said they had no issues with objection as the services could run without issue given high numbers of staff. - Others believed that objection should not be allowed, particularly in rural areas where it may threaten access to care. - Despite this, all supported the right to objection if not comfortable to provide. | | 5 |
| [11] | McLeod, 2021 | United States  227 providers  Obstetricians, Non-Obstetricians | Comprehensive abortion service | | Qualitative  Survey  Analysis guided by Crabtree and Miller (1999) | | - The providers believed that pregnancy experiences had made them more pro-choice, contrasting with prominent cultural discourse. - Experience of pregnancy had also increased levels compassion towards patients and improved the therapeutic bond between provider and patient. - Some said that personal experiences of pregnancy loss made it difficult to provide. Others said that looking at fetal remains was more challenging following pregnancy. - Many believed that being an abortion provider made them a better parent. | | 3 |
| [12] | Persson, 2021 | Bangladesh  24 providers  Key Informants, Doctors, Paramedics | Comprehensive abortion service | | Qualitative  Interviews  Content analysis | | - The availability of and access to comprehensive abortion care in Cox’s Bazar was limited by restrictive abortion policies and the lack of appropriate space. - Though training has helped to improve providers’ competence and confidence in delivering safe abortion care, their knowledge of the law was low. - Providers’ perceptions of abortion among Rohingya women influenced the care they provided. | | 5 |
| [13] | Rasmussen, 2021 | United States  19 providers  Family Medicine Physicians, Nurses, Nurse Midwifes, Administrators | Comprehensive abortion service | | Qualitative  Interviews  Analysis described but not named | | - The providers expressed support for pharmacy led abortion care services, as it would be able to normalise abortion, reduce implementation barriers in primary care, and expand access to safe abortion care. - Challenges to address included restrictive regulations on mifepristone, federal funding restrictions on abortion, concerns about unsupervised medical abortion, and the potential for objection among pharmacists. | | 4 |
| [14] | Simmonds, 2021 | United States  8 providers  Nurses | Comprehensive abortion service | | Qualitative  Interviews  Analysis described but not named | | - Nurses believed that providing early abortion care is generally a positive experience and that having the opportunity to connect with their patients emotionally was gratifying. - They also reflected positively on their ability to resolve their patients’ needs and believed that they could support their beliefs by providing abortion care. - They received support within the workplace and their personal lives. | | 5 |
| **Ref** | **Author, Year** | **Country**  **Sample Size**  **Professions** | | **Procedures Provided ^a^** | | **Methodology**  **Data Collection Method**  **Analysis** | | **Key Findings** | **MMAT Score ^b^** |
| [15] | Zwerling, 2021 | United States  15 providers  Nurses | Abortion for fatal fetal abnormalities | | Qualitative  Interviews  Thematic analysis | | - The nurses emotionally, logistically, and morally struggled with abortion for fetal abnormalities. Challenges included the emotionally intense work of abortion, feelings of incompetence in providing emotional support, ethical conflicts, and value judgments. - Despite challenges, the nurses perceived a duty to their patients. | | 3 |
| [16] | Aborigo, 2020 | Ghana  12 providers  Midwife, Obstetrician, Medical Practitioner, Medical Assistant, Community Health Officer, Pharmacist, Chemical Seller, Traditional Birth Attendant | | Comprehensive abortion service | | Qualitative  Interviews  Thematic analysis | | - Providers in Ghana experience stigma related to their abortion work, as Ghanian culture holds strong opposition to abortion. - Providers believed that conscientious objection in the face of stigma was a key barrier for the services. - Providers valued the opportunity to complete values clarifications workshops and to meet with a mentor. | 3 |
| [17] | Ewnetu, 2020 | Ethiopia  30 providers  Nurses, Midwives, Obstetricians, GPs, Public health specialists | | Comprehensive abortion service | | Qualitative  Interviews  Framework analysis | | - Many providers were challenged by the idea of the fetus’ right to life. - Views on when life begin differed, some believed at conception and others believed that it developed over the pregnancy. - Many settled this conflict by emphasising a religious norm or professional duty to provide care and respect their patients’ decisions. | 5 |
| [18] | Hasselbacher, 2020 | United States  31 providers  Ob/Gyn, Midwife/Nurse, Family Physicians, other physicians, ethicists, administrators, chaplains | | Comprehensive abortion service | | Qualitative  Interviews  Thematic content approach | | - Providers in Catholic hospitals felt highly restricted in their ability to provide abortion care for any reason. - Providers in Protestant ethos hospitals experiences restrictions in providing “elective” abortion care. - Some in these hospitals lamented their inability to provide care for their patients as they were trained to do. - Providers in secular hospitals reported few barriers to their provision of care. | 5 |
| **Ref** | **Author, Year** | **Country**  **Sample Size**  **Professions** | | **Procedures Provided ^a^** | | **Methodology**  **Data Collection Method**  **Analysis** | | **Key Findings** | **MMAT Score ^b^** |
| [19] | Mavuso, 2020 | South Africa  4 providers  Nurses, Counsellors | | Comprehensive abortion service | | Qualitative  Interviews  African feminist approach into Taylor et al. narrative-discursive method | | - Providers in South Africa experienced stigma related to their abortion work. - The providers explained their decision to do abortion work through the socially affirming hero canonical narrative, helping them to reframe abortion work from stigmatised to honourable work that adds positively to society. | 2 |
| [20] | Maxwell, 2020 | United Kingdom  20 providers  Nurses, clinical support workers, doctors, sonographer, management | | Comprehensive abortion service | | Qualitative  Interviews  Thematic analysis | | - Providers in the United Kingdom experienced resistance and even hostility from some non-providing colleagues. - They experienced stigma related to their abortion work, both among colleagues and outside the workplace. Many chose to limit disclosing their work to avoid stigma outside the workplace. - Providers viewed their involvement in abortion care positively, emphasising that they provide a normal and important service. | 4 |
| [21] | Mosley, 2020 | 152 providers  Latin America (7 unnamed countries), Africa (3 unnamed countries)  Jobs not given | | Unknown | | Quantitative (Longitudinal non-randomized)  Survey, workshop  Statistical analyses | | - Providers across Latin America and Sub-Saharan Africa experience stigma related to their abortion work. - In Africa, level was stigma was positively related to risk of burnout. - Level of stigma in both regions decreased after participating in a provider support intervention. | 4 |
| [22] | Nkosi, 2020 | South Africa  29 providers  Facility and Quality Managers, Nurses, Midwives | | Comprehensive abortion service | | Quantitative  Survey  Statistical analyses | | - Providers in South Africa noted a range of infrastructural challenges to providing care, including appropriate space to provide, air conditioning, staff facilities, necessary equipment, adequate staffing numbers, and the opportunity to work in other areas. | 4 |

| **Ref** | **Author, Year** | **Country**  **Sample Size**  **Professions** | **Procedures Provided ^a^** | **Methodology**  **Data Collection Method**  **Analysis** | **Key Findings** | **MMAT Score ^b^** |
| --- | --- | --- | --- | --- | --- | --- |
| [23] | Påfs, 2020 | Rwanda  52 providers  Physicians, Midwives, Nurses | Comprehensive abortion service | Qualitative  Interviews, focus groups  Thematic analysis | - Many were uncertain about the legal status of induced abortion in Kenya after the 2012 constitutional amendments. - Abortion is highly stigmatised in Kenya, and providers try to avoid becoming associated with the procedure. - Many feared litigation or liability if they unknowingly provided against the current legislation. | 5 |
| [24] | Power, 2020 | Ireland  10 providers  Maternal fetal medicine subspecialists | Abortion for fatal fetal abnormalities | Qualitative  Interviews  Thematic analysis | - MFM sub-specialists in Ireland feared interpreting the new legislation regarding fatal fetal abnormalities incorrectly. - All underlined the importance of supportive colleagues, though many had experience with colleagues who did not support the services. - They discussed the need to tailor care to the needs of each patient. - Almost all experienced internal conflict when providing care, and the provision of feticide was highlighted as particularly challenging. | 5 |
| [25] | Ramón Michel, 2020 | Argentina  185 providers  GP/Family Practitioners, Obstetricians, Nurses, Social Workers, Psychologists | Comprehensive abortion service | Mixed Methods  Survey, Interviews  Statistical analyses, Thematic analyses | - Many believed that colleagues have misused the right to conscientiously object to abortion care. - High levels of objection left providers feeling isolated. They also noted large issues relating to delays in care and stigma. - Many believed that there should be greater regulations on objection. | 5 |
| [26] | Teffo, 2020 | South Africa  30 providers  Nurses | Comprehensive abortion service | Qualitative  Interviews  IPA | - Providers in South Africa experienced stigma related to their abortion work. - To minimise stigma, many chose not to discuss their abortion work, though silence constituted an emotional burden. - Providers sought informal and formal support. - Some developed feelings of “numbness” to providing, particularly in response to later-gestation and repeat abortions. - Many used religion to cope with the challenging aspects of care, saying that abortion was a calling and that they got relief from prayer. | 3 |

| **Ref** | **Author, Year** | **Country**  **Sample Size**  **Professions** | **Procedures Provided ^a^** | **Methodology**  **Data Collection Method**  **Analysis** | **Key Findings** | | **MMAT Score ^b^** |
| --- | --- | --- | --- | --- | --- | --- | --- |
| [27] | Zareba, 2020 | Poland  94 providers  Midwives | Comprehensive abortion service | Quantitative (Cross-sectional observational)  Survey  Statistical analyses | | - Moral doubts were common among midwives in Poland who provided abortion care, particularly when in contact with the fetus. - They also reported a lack of supports available to deal with the challenging aspects of care. | 1 |
| [28] | Czarnecki, 2019 | United States  50 providers  Nurses, Anaesthesiologists, Maternal Fetal Medicine subspecialists, Obstetrics residents | Comprehensive abortion service | Qualitative  Interviews  Analysis described but not named | - Providers were challenged by the realities of abortion care, particularly when performed in the second trimester. - Many drew distinctions between “elective” and “worthy,” “medically indicated” cases, believing that it was easier to justify their involvement with the latter. - Providers believed that their duty to provide care was more important than their own beliefs. | | 2 |
| [29] | Fernández, 2019 | Argentina  27 providers  GPs, Gynaecologists, Social workers, Psychologists, Sociologist, Paediatrician, Pharmacist | Comprehensive abortion service | Qualitative  Interviews  Analysis described but not named | - Providers in Argentina experience stigma related to their abortion work. - They said that healthcare staff helped to design the abortion legislation, helping to improve clarity and the services. - Providers worked together to support each other following liberalisation. | | 3 |
| [30] | McLean, 2019 | Ethiopia  42 providers  Doctors, Nurses, Nurse/Midwives, Health Officers, Medicine Students, Pharmacist | Comprehensive abortion service | Qualitative  Interviews, Focus Groups  Content analysis | - Providers in Ethiopia experienced a gap between the laws and their clinical experience, for example refusing care could result in the woman’s death, some patients lie to access care, and it wasn’t always clear what circumstances the law covered. - Many feared that they could provide abortion against the law. - Providers in Ethiopia experienced stigma related to their abortion work. - Despite challenges, they believed abortion helped to save women’s lives. | | 4 |

| **Ref** | **Author, Year** | **Country**  **Sample Size**  **Professions** | **Procedures Provided ^a^** | **Methodology**  **Data Collection Method**  **Analysis** | **Key Findings** | | **MMAT Score ^b^** |
| --- | --- | --- | --- | --- | --- | --- | --- |
| [31] | Oelhafen, 2019 | Switzerland  10 providers  Midwives, Nurse, Physician | Comprehensive abortion service | Qualitative  Interviews  Thematic analysis | - Many providers found abortion work difficult if they did not understand the persons decision to access care. - It is difficult to maintain moral distance from the decision to end the pregnancy when actively involved in the procedure. | | 5 |
| [32] | Seewald, 2019 | United States, Latin America (unnamed country), Africa (unnamed country) | Unknown | Qualitative  Workshop  Thematic analysis | - Using the stories of providers from three different regions who participated in a support intervention, this study explores the ways in which stigma contributes, both directly and indirectly, to abortion complications, makes them more difficult to treat, and impacts the ways in which they are resolved. | | 1 |
| [33] | Cárdenas, 2018 | Uruguay  20 providers  Physicians, Midwives, Social Workers, Psychiatrist | Comprehensive abortion service | Qualitative  Interviews  Thematic analysis | - Providers believed that liberalisation helped to destigmatise abortion in Uruguay, even if stigma still existed. - Many experienced issues related to high levels of conscientious objection. - Some disagreement about the legislation remained, particularly in rural areas where providers felt unsupported. - They believed that abortion was not used as a method of contraception. - They said that medical abortion care was easier to provide than surgical. | | 3 |
| [34] | De Zordo, 2018 | Italy and Spain  77 providers  Obstetricians | Comprehensive abortion service | Mixed methods  Interviews, survey  Analysis not discussed | - Obstetricians in Italy and Spain experience stigma related to their work. - They made moral distinctions between “good,” medically indicated abortions and “bad” abortions. Repeat abortions were least acceptable. - Providers believed that abortion was dirty work, and most disliked and did not provide surgical methods of care. - Despite challenges, most believed that abortion is “necessary.” | | 1 |
| [35] | Greenberg, 2018 | United States  20 providers  Obstetricians | Comprehensive abortion service | Qualitative  Interviews  Template analysis | - Most felt comfortable in providing medical care, though felt that surgical was more technically and emotionally demanding. - Institutional and technical barriers also disrupted intentions to provide abortion care. - Most believed that abortion training was valuable. | | 2 |
| [36] | Janiak, 2018 | United States  136 providers  Nurses, Medical assistants, counsellors | Comprehensive abortion service | Quantitative (Cross-sectional observational)  Survey  Statistical analyses | | - Providers in Massachusetts experienced related to their abortion work. - Stigma was higher among those who regularly attended religious events compared to those who didn’t. Stigma was lower among counsellors compared to other professions. - Every clinic experienced regular protests, while hospitals did not. | 4 |
| **Ref** | **Author, Year** | **Country**  **Sample Size**  **Professions** | **Procedures Provided ^a^** | **Methodology**  **Data Collection Method**  **Analysis** | **Key Findings** | | **MMAT Score ^b^** |
| [37] | Martin, 2018 | United States  315 providers  Physicians, Nurses, Medical Assistants, Managers, Advanced Care, Counsellors, Senior Leaders | Comprehensive abortion service | Quantitative (Longitudinal non-randomized)  Survey  Statistical analyses | - Providers from across the United States experience stigma related to their abortion work. - Correlation analysis revealed that level of stigma was positively correlated with providers’ risk of burnout. - Three quarters reported pride in their practice despite challenges. | | 4 |
| [38] | Puri, 2018 | Nepal  106 providers  Obstetrician, Physicians, Nurses, Auxiliary nurses, midwives, counsellors | Comprehensive abortion service | Quantitative (Cross-sectional observational)  Survey  Statistical analyses | - The most common barriers to the provision of care included irregular supply of medication, lack of trained providers and staff, and a lack of space to provide care. - Providers believed that the needs of the patients are paramount to the beliefs of the provider, and that every woman should have the right to access care. | | 4 |
| [39] | Britton, 2017 | United States  27 providers  Obstetricians, Family Medicine Physicians, Physician Assistants, Nurses | Early abortion (Medical and surgical methods) | Qualitative  Interviews  Grounded theory | - The Women’s Right to Know Act was perceived to impact the standard of care. - Many believed that the law further stigmatised and marginalised abortion care by implying that providers needed strict guidance on how to counsel patients. - Providers motivated to continue practice to help patients and expressed pride in their ability to overcome challenges. | | 5 |
| [40] | Dawson, 2017 | Australia  8 providers  General practitioners | Early abortion (Medical methods only) | Qualitative  Interviews and focus groups  Thematic analysis | - Many GPs discussed fear of stigma and repercussion if they discussed or promoted their abortion work. Most experienced stigma. - Rural GPs felt isolated, with many not knowing a providing colleague. - Most GPs noted gaps in or the absence of peer support systems. - GPs experienced challenges when trying to refer a patient for additional supports/care. | | 5 |

| **Ref** | **Author, Year** | **Country**  **Sample Size**  **Professions** | **Procedures Provided ^a^** | **Methodology**  **Data Collection Method**  **Analysis** | **Key Findings** | **MMAT Score ^b^** |
| --- | --- | --- | --- | --- | --- | --- |
| [41] | Martin, 2017 | United States  96 providers  Counsellor, Nurse, Physicians, Recovery room assistants, managers, surgical assistants, prep/clean-up, other | Comprehensive abortion service | Qualitative  Workshop  Analysis described but not named | - Many providers from across the United States perceived discomfort in aspects of their abortion work. Discomfort included judging some patients, experiencing moral doubt, fulfilling anti-abortion stereotypes, and difficult emotional reactions to the fetal remains. - In response to these challenges, providers censor themselves when talking about their work in fear that sharing information could put access to abortion care at risk. - They also felt disconnected from pro-choice messaging, and that their experience of care was not represented. - Providers said that discussing difficult aspects of their work with colleagues was a benefit. | 2 |
| [42] | Mauri, 2017 | Italy  24 providers  Nurses, Midwives | Comprehensive abortion service | Qualitative  Interviews  Phenomenological analysis | - Nurses and midwives in Italy said that it may be hard to provide care for foreign women, young women, and in cases of repeat abortion. Many also found the psychological component of care demanding. - Many said they have become “mechanistic” to deal with the challenging aspects of abortion care. - Providers called for improvements to professional training. - Providers preferred medical abortion to previous surgical methods. - All discussed positive and negative emotions linked with cases they have cared for. | 3 |
| [43] | Purcell, 2017 | United Kingdom  37 providers  Nurses, Doctors, Clinical support workers, Sonographers | Comprehensive abortion service | Qualitative  Interviews  Thematic framework analysis | - Providers discussed the high levels of emotional labour involved in abortion work and the need to calm and comfort patients. - They said that providing care at increased gestations is challenging. - They also said that standardised clinic time has a hindrance to care. - Abortion care also involves a large amount of hands-on body work, which made some feel more responsible for the fetal demise. | 5 |

| **Ref** | **Author, Year** | **Country**  **Sample Size**  **Professions** | **Procedures Provided ^a^** | **Methodology**  **Data Collection Method**  **Analysis** | **Key Findings** | **MMAT Score ^b^** |
| --- | --- | --- | --- | --- | --- | --- |
| [44] | Teffo, 2017 | South Africa  30 providers  Nurses | Comprehensive abortion service | Mixed methods  Interviews, field notes  IPA, Statistical analyses | - Nurses discussed rewarding aspects of providing abortion care, such as preventing complications and unwanted/teenage pregnancies. - They had negative experiences with their colleagues, including stigma and a lack of support from fellow nurses, doctors, and management. - The services had a lack of necessary equipment, medication, and staff, and were situated in an unsuitable space. - The nurses also discussed the emotional burden of providing, including feelings of isolation and the emotional trauma of abortion. | 4 |
| [45] | Aniteye, 2016 | Ghana  36 providers  Obstetricians, Nurses, Midwives, Pharmacists | Early abortion (Medical and surgical methods) | Qualitative  Interviews  Framework analysis | - Abortion is heavily stigmatised in Ghanian culture and this stigma impacts providers, e.g. negative reflections on their involvement, stigmatising interactions outside of the clinic, and lack of recognition for abortion work. - Some perceived the Ghanian law to be ambiguous, which led some to believe that there is a legal risk in providing. - Majority believed women should have access to care. | 4 |
| [46] | Debbink, 2016 | United States  79 providers  Counsellors, Nurses, Physicians, Recovery room assistants, Managers, Surgical Assistants, Prep/clean-up | Comprehensive abortion service | Qualitative  Workshop, survey  Analysis described but not named | - Providers from across the United States experience stigma related to their abortion work. - Providers valued the opportunity to discuss stigma and other challenging aspects of their work in a supportive and safe space with their colleagues. - They valued the opportunity to participate in the Providers Share Workshop | 2 |
| [47] | Fay, 2016 | United Kingdom  10 providers  Maternal fetal medicine subspecialists | Feticide & abortion for fatal fetal abnormalities | Qualitative  Interviews  IPA | - Providers believed that feticide is a difficult procedure that is both emotionally and technically challenging to perform. - Many said that they hide this part of their work in fear of stigma. - Despite being difficult, they believed that feticide was a part of their job. - They felt that the law was ambiguous in how it defined fatal fetal abnormalities, and providers discussed fear of litigation. - They believed that working in a multi-disciplinary team helped to reduce doubts about their interpretation. | 5 |
| **Ref** | **Author, Year** | **Country**  **Sample Size**  **Professions** | **Procedures Provided ^a^** | **Methodology**  **Data Collection Method**  **Analysis** | **Key Findings** | **MMAT Score ^b^** |
| [48] | Larsson, 2016 | Sweden  13 providers  Doctors, Midwives | Comprehensive abortion service | Qualitative  Interviews  Thematic analysis | - The providers believed that immigrant women have additional needs in abortion care but didn’t want to discuss this openly. - Many felt that more conceptive counselling was needed. - To provide equitable access to care, the providers called for extra resources, such as when translation services and extra time. | 5 |
| [49] | Purcell, 2016 | United Kingdom  25 providers  Nurses, Doctors | Comprehensive abortion service | Qualitative  Interviews  Thematic framework analysis | - Providers believed that abortion should not be used as a method of contraception, with many viewing repeat abortions negatively. - Contraception provided at abortion helped to reduce discomfort. | 5 |
| [50] | Yang, 2016 | Taiwan (China)  22 providers  Nurses | Comprehensive abortion service | Qualitative  Interviews  Content Analysis | - Nurses in Taiwan discussed the need to concealing negative or difficult emotions when providing abortion care. - They felt as though they could not conscientiously object to care. - They experienced mental unease when providing care and tried to treat the fetal remains with respect to avoid negative consequences. | 5 |
| [51] | Black, 2015 | Australia  22 providers  Doctors | Abortion for fatal fetal abnormalities after 20 weeks | Qualitative  Interviews  Thematic Analysis | - Doctors believed that regional laws were too restrictive, forcing women with pregnancies affected by fatal fetal abnormalities to travel for care. - All experienced delays with ethics committees, resulting in undue stress for women trying to access care. | 2 |
| [52] | Mauri, 2015 | Italy  17 providers  Midwives | Abortion for fatal fetal abnormalities after 16 weeks | Qualitative  Interviews  Phenomenological hermeneutic analysis | - Midwives in Italy said that abortion care was emotionally difficult and that they suffer with their patients. Some experienced anger when involved abnormalities are compatible with life. - They said that had good levels of peer support, but that management were lacking in training and organisation. - They believed that providing became easier with experience. | 5 |

| **Ref** | **Author, Year** | **Country**  **Sample Size**  **Professions** | **Procedures Provided ^a^** | **Methodology**  **Data Collection Method**  **Analysis** | **Key Findings** | **MMAT Score ^b^** |
| --- | --- | --- | --- | --- | --- | --- |
| [53] | McLemore, 2015a | United States  25 providers  Nurses | Comprehensive abortion service | Qualitative  Interviews  Qualitative description & thematic analysis | - Nurses in the United States had to tack between their personal and professional perspectives when providing care, helping to protect themselves against difficult emotions and to conceal emotions from patients. - Many experienced a lack of support and stigma from some non-providing nursing colleagues and doctors. - They differentiated between knowing how to provide care and knowing why women choose to access care, noting the importance of supporting each woman as an individual. | 5 |
| [54] | McLemore, 2015b | United States  16 providers  Nurses | Comprehensive abortion service | Qualitative  Interviews  Thematic analysis influenced by Grounded Theory methods | - Exposure to abortion care through education and/or employment is important to recruiting new nurses to provide abortion care. - To remain in abortion work, many nurses highlighted the need to be flexible in their attitudes towards abortion work and to grow their own skills in the absence of professional development opportunities. - To progress their careers in abortion care, nurses called for more activities to legitimise abortion care as a distinct field, such as conferences and continuing education. | 2 |
| [55] | Mercier, 2015 | United States  31 providers  Obstetrician, Family Physician, Nurses, Physician assistant, counsellor, administrator | Comprehensive abortion service | Qualitative  Interviews  Grounded theory | - Providers viewed the Women’s Right to Know in negative terms, namely that it sought to indirectly restrict access to abortion care. - The law had several negative impacts on patients and providers. - The providers believed they had been able to adapt and to normalise their practice despite challenges created by the law. | 3 |
| [56] | Potdar, 2015 | India  19 providers  Gynaecologists | Comprehensive abortion service | Qualitative  Interviews  Analysis not described | - The Pre-Conception and Pre-Natal Diagnostic Techniques Act (2003) in India has made access to legal abortion care more difficult. - Due to fear of criminal charges related to sex-selective abortion, many gynaecologists refuse to provide care to some women. - Many providers believed that the act was being used against them. | 1 |

| **Ref** | **Author, Year** | **Country**  **Sample Size**  **Professions** | **Procedures Provided ^a^** | **Methodology**  **Data Collection Method**  **Analysis** | **Key Findings** | **MMAT Score ^b^** |
| --- | --- | --- | --- | --- | --- | --- |
| [57] | Strefling, 2015 | Brazil  19 providers  Nurses | Comprehensive abortion service | Qualitative  Interviews  Discourse of the collective subject | - Some nurses believed that care was overly clinical, given stigma around care and a lack of contact with patients. - Others considered care to be appropriate, regardless of the indication for the abortion, highlighting the importance of empathetic and compassionate care. | 2 |
| [58] | Andersson, 2014 | Sweden  21 providers  Midwives, Midwifery Students, Nurses | Second trimester medical abortion | Qualitative  Interviews  Thematic content analysis | - Midwives/Nurses felt powerlessness to prevent pain during the abortion. - They regarded handling the fetus as the most challenging aspect of care. - Some felt that providing abortion care contradicted their duty to preserve life, while others believed that abortion was used as contraception. - They valued opportunities to talk about work with their colleagues. - It was important to develop maturity, to conceal emotions, and to support women through the abortion experience. | 5 |
| [59] | Gwangwa, 2014 | South Africa  10 providers  Nurses | Comprehensive abortion service | Qualitative  Interviews  Tesch’s method of qualitative analysis | - Nurses in South Africa noted a shortage of human and material resources, which was a source of stress and frustration for many. - They also experienced stigma related to their abortion work. - Many struggled with personal beliefs against abortion. - They noted a lack of collegial and managerial support. | 3 |
| [60] | Martin, 2014a | United States  55 providers  Physicians, Nurses, Counsellors, Managers, Recovery Room Assistants, Surgical Assistants, Preparation/Clean-up, Other | Comprehensive abortion service | Quantitative (Longitudinal non-randomised)  Survey  Statistical analyses | - Providers from across the United States experience stigma related to their abortion work. - Surgical assistants reported significantly lower levels of stigma compared to their colleagues in other categories. - Concerns related to disclosing their abortion work were common. | 3 |

| **Ref** | **Author, Year** | **Country**  **Sample Size**  **Professions** | **Procedures Provided ^a^** | **Methodology**  **Data Collection Method**  **Analysis** | **Key Findings** | **MMAT Score ^b^** |
| --- | --- | --- | --- | --- | --- | --- |
| [61] | Martin, 2014b | United States  79 providers  Physicians, Nurses, Counsellors, Medical Assistants, Administrative Staff | Comprehensive abortion service | Quantitative (Longitudinal non-randmized)  Survey  Statistical analyses | - Providers from across the United States experience stigma related to their abortion work. - Providers in hospital-based practice has lower experience of stigma compared to providers in free-standing clinics. - As stigma increased, providers satisfaction with their work decreased. | 4 |
| [62] | Parker, 2014 | Canada  10 providers  Nurses | Abortion for fatal fetal abnormalities | Qualitative  Interviews  Content analysis | - To cope with the challenging aspects of care, nurses highlighted the importance of receiving support from mentors and colleagues. - They also developed personal strategies to cope with the challenging aspects, such as affirming the importance of work and developing a therapeutic relationship with their patients. - They called for more staff members to help reduce the workload and for more opportunities to learn about abortion. | 4 |
| [63] | Aniteye, 2013 | Ghana  43 providers  Obstetricians, Midwives, Pharmacists, Other | Comprehensive abortion service | Qualitative  Interviews  Framework analysis | - Providers in Ghana experience stigma related to their abortion work, as Ghanian culture holds strong opposition to abortion. - Stigma was a key barrier for obstetricians to become involved in care. - Many people in management positions opposed the services, creating challenges for the providers. - Some talked about internal conflict related to their religious beliefs and their provision of care, though believed that abortion is a necessary service. | 1 |
| [64] | Christensen, 2013 | Denmark  10 providers  Midwives | Second and third trimester fetal abnormality | Qualitative  Interviews  Grounded theory | - Midwives highlighted the challenges of providing, particularly in acknowledging the grief of the patients and partner and when handling the fetal remains. - Experience helps midwives to cope with the challenging aspects. - Midwives provide care because they believe it is the right thing to do. | 3 |
| [65] | Dressler, 2013 | Canada  20 providers  Physicians | Early abortion (Surgical methods) | Qualitative  Interviews  Thematic analysis | - Many rural providers discussed fear of stigma and repercussion if they discussed or promoted their abortion work. Most experienced stigma. - Rural providers felt isolated from other providers and felt stuck. - They also experienced difficulties in referring patients for additional support/care. | 5 |

| **Ref** | **Author, Year** | **Country**  **Sample Size**  **Professions** | **Procedures Provided ^a^** | **Methodology**  **Data Collection Method**  **Analysis** | **Key Findings** | **MMAT Score ^b^** |
| --- | --- | --- | --- | --- | --- | --- |
| [66] | Mizuno, 2013 | Japan  255 providers  Nurses, Midwives | Comprehensive abortion service | Quantitative (Cross-sectional observational)  Survey  Statistical analyses | - Nurses and midwives said that the most stressful aspect of abortion work related to the fetus. - Number of first-trimester abortions handled was positively related to burnout. | 4 |
| [67] | Norman, 2013 | Canada  39 providers  Obstetrician, Family physician, | Comprehensive abortion service | Quantitative (Cross-sectional observational)  Survey  Statistical analyses | - Providers working in rural Canada experienced stigma related to their abortion work, high levels of stigma had forced many peers to stop providing. - They also experienced difficulties in referring patients for additional support/care and when trying to access external resources. | 5 |
| [68] | Turk, 2013 | United States  105 providers  Family Planning Subspecialists | Second-trimester abortion (surgical methods) | Quantitative (Cross-sectional observational)  Survey  Statistical analyses | - Providers in more abortion-restrictive regions were four times more likely to report a personal main barrier (such as concern for safety) than other types of main barriers. - Providing D&Es in a hospital operating room was associated with 2.8 times higher odds of reporting an institutional or co-worker main barrier. - High-volume D&E practice was associated with three times lower odds of reporting an institutional/co-worker main barrier. | 4 |
| [69] | Harries, 2012 | South Africa  19 providers  Obstetricians, Doctors, Nurses, Senior Hospital Managers | Second-trimester abortions (surgical methods) | Qualitative  Interviews  Thematic analysis | - Providers believed abortion in the second trimester is more traumatic than in the first trimester, given that the fetus is “formed”. - They preferred medical abortion, saying that the dilation and evacuation procedure elicited more physical and emotional responses. - Barriers to providing abortion in the second trimester included lack of infrastructural support and stigma. | 5 |
| [70] | Möller, 2012 | Nepal  15 providers  Obstetricians, Doctors, Nurses | Comprehensive abortion service | Qualitative  Interviews  Grounded theory (constant comparative method) | - Though abortion stigma exists in Nepal, providers had little experience. - They reflected positively on liberalisation, believing that they are making a positive contribution to society. - Some experienced conflicts about potential misuse and illegal sex-selection. - The providers were proud of their work but acknowledged that they had more to do to ensure access to care for all in Nepal. | 3 |

| **Ref** | **Author, Year** | **Country**  **Sample Size**  **Professions** | **Procedures Provided ^a^** | **Methodology**  **Data Collection Method**  **Analysis** | **Key Findings** | **MMAT Score ^b^** |
| --- | --- | --- | --- | --- | --- | --- |
| [71] | Perrin, 2012 | Switzerland  77 providers  Doctors, Nurses, Midwives, Psychologists | Comprehensive abortion service | Qualitative  Interviews  Customary approach | - The providers had varying opinions on the care of minors without parental consent. - Some believed that providing abortion was a “necessary evil,” admitting that it was difficult work but a socially important service. | 1 |
| [72] | Puri, 2012 | Nepal  35 providers  Obstetricians, Nurses, Health Assistant, Abortion Counsellor, Hospital Administrator | Comprehensive abortion service | Qualitative  Interviews  Thematic approach (described but not referenced) | - Providers in Nepal viewed abortion liberalisation positively, as it lowered the number of complications and prevented death. - Some providers held negative attitudes about the reasons for some abortions, particularly unmarried women and repeat abortion. | 5 |
| [73] | Contreras, 2011 | Mexico  64 providers  Obstetrician, Nurses, Social Workers, Physicians, Receptionists, Anaesthesiologists, other | Comprehensive abortion service | Qualitative  Interviews  Analysis described but not named | - Providers said that there were high levels of confusion when the legislation was first introduced, though this was quickly resolved. - Providers reported confusion around conscientious objection and believed that high levels of objection created barriers to care. - They believed that management failed to provide adequate space, medication, equipment, training, and personnel for the services. - Providers worked with the support of the Ministry of Health to overcome many of these issues in the first year of the services. | 1 |
| [74] | Halldén, 2011 | Sweden  10 providers  Midwives | Comprehensive abortion service | Qualitative  Interviews  Phenomenological hermeneutic analysis | - Midwives acknowledged challenges of providing abortion care for minors, particularly when they attended without a partner or parent. - They felt that most minors were nonchalant about abortion and felt powerless to change their views. - They believed that respecting other’s views about abortion and when life begins was a prerequisite for providing abortion care. | 5 |

| **Ref** | **Author, Year** | **Country**  **Sample Size**  **Professions** | **Procedures Provided ^a^** | **Methodology**  **Data Collection Method**  **Analysis** | **Key Findings** | **MMAT Score ^b^** |
| --- | --- | --- | --- | --- | --- | --- |
| [75] | Harris, 2011 | United States  17 providers  Doctors, counsellors, managers, nurses, medical/surgical assistants | Comprehensive abortion service | Qualitative  Workshop  Analysis described but not named | - Providers across the United States experienced stigma related to their abortion work. - Inside their workplace, providers experienced stigma from patients and co-workers. - Outside the workplace, they experienced stigma from friends, family members, strangers, and public discourse. - In response to stigma, many chose to with-hold disclosing their work. | 2 |
| [76] | Lamichhane, 2011 | Nepal  35 providers  Physicians, Nurses, Counsellors, Administrators | Comprehensive abortion service | Qualitative  Interviews  Thematic analysis | - Providers in Nepal viewed abortion liberalisation positively but held concerns about the ban on sex-selective procedures. - Acknowledging the cultural benefits wanting male children, providers worried about litigation if they unknowingly provided care. | 5 |
| [77] | Lindström, 2011 | Sweden  40 providers  Obstetricians, Midwives | Comprehensive abortion service | Qualitative  Focus groups  Content analysis | - Gynaecologists believed that changing procedures made provision of care easier, though were still challenged by repeat abortions. - Midwives said that late-gestation care was challenging, particularly if the procedure is not medically indicated. - All believed that abortion was a necessary and socially important service. | 5 |
| [78] | Lipp, 2011 | United Kingdom  5 providers  Nurses, Midwives | Comprehensive abortion service | Qualitative  Interviews  Grounded theory | - Nurses and midwives highlighted the importance of being non-judgmental, and of concealing the judgments they do have. - They view first trimester abortions as significantly easier than later. - They needed to tailor their care to the needs of each patient. - They could self-preserve by “switching off” when providing care. | 5 |
| [79] | Mizuno, 2011 | Japan  11 providers  Midwives | Comprehensive abortion service | Qualitative  Interviews  Thematic analysis | - Thinking about the fetus was a prominent challenge for midwives assisting in abortion care in Japan and found it difficult to care for women accessing abortion for different reasons simultaneously. - Midwives needed to establish control over their emotions. - They were challenged by the duality of preserving life in their midwifery role and performing abortions, ultimately deciding that it should be the woman’s choice to access care. | 5 |

| **Ref** | **Author, Year** | **Country**  **Sample Size**  **Professions** | **Procedures Provided ^a^** | **Methodology**  **Data Collection Method**  **Analysis** | **Key Findings** | **MMAT Score ^b^** |
| --- | --- | --- | --- | --- | --- | --- |
| [80] | O’Donnell, 2011 | United States  14 providers  Physicians, Midwives, Nurses, Social worker | Comprehensive abortion service | Qualitative  Interviews  Unnamed analysis described | - Providers in the United States experienced stigma related to their abortion work. - Stigma occurred inside the workplace, with patients and colleagues, as well as outside the workplace, with colleagues, family, friends, and strangers. - To resist stigma, providers reframed their work from stigmatised work to noble work that adds positively to society. | 2 |
| [81] | Freedman, 2010 | United States  3 providers  Obstetricians | Comprehensive abortion service | Qualitative  Interviews  Grounded theory | - Obstetricians said that their workplace restricted their ability to provide abortion care, either made clear before or after they were hired. - Obstetricians experienced stigma about their work among colleagues. - Some said that the threat of violence was the biggest deterrent to providing abortion care. | 3 |
| [82] | Gallagher, 2010 | United Kingdom  9 providers  Nurses, Midwives | Comprehensive abortion service | Qualitative  Interviews  Thematic analysis | - Nurses in the United Kingdom experienced stigma related to their abortion work. To cope with stigma, they with-hold disclosure and affirmed that abortion is the woman’s decision. - They struggled with later-gestation abortion. - They supported women but found parts of their work difficult. - Support from their colleagues was considered essential. | 3 |
| [83] | Lipp, 2010 | United Kingdom  12 providers  Nurses, Midwives | Comprehensive abortion service | Qualitative  Interviews  Grounded theory | - Nurses and midwives in the UK conceded that they sometimes judged women, despite affirming that providers need to be non-judgmental. Repeat abortions were an example. - They used maxims and empathy to conceal their judgments | 5 |
| [84] | Mamabolo, 2010 | South Africa  3 providers  Nurses | Comprehensive abortion service | Qualitative  Interviews  Tesch’s method of qualitative analysis | - Nurses in South Africa experienced stigma related to their abortion work. - Nurses experienced negative emotions when providing abortion care. - All dealt with challenges by talking about their work and seeking peer support. The nurses reported low levels of managerial support. | 4 |

| **Ref** | **Author, Year** | **Country**  **Sample Size**  **Professions** | **Procedures Provided ^a^** | **Methodology**  **Data Collection Method**  **Analysis** | **Key Findings** | **MMAT Score ^b^** |
| --- | --- | --- | --- | --- | --- | --- |
| [85] | Nicholson, 2010 | United Kingdom  7 providers  Nurses | Comprehensive abortion service | Mixed methods  Interviews, survey  IPA, statistical analyses | - To help cope with the challenging aspects of abortion care, the nurses talked about developing unconditional acceptance for all patients, talking about their experiences with supportive colleagues, and emphasising the psychological aspect of abortion care. - Challenging aspects of abortion care included perceived misuse of the services, juggling the contrasting needs of patients, handling the fetal remains, and the heavy workload. - The nurses also drew upon personal life experiences of abortion and pregnancy loss to support patients. | 2 |
| [86] | Graham, 2009 | United Kingdom  23 providers  Obstetricians, Midwives | Feticide & abortion for fatal fetal abnormalities | Qualitative  Interviews  Interpretative qualitative approach | - Providers believed that feticide is a difficult procedure that is both emotionally and technically challenging to perform. - Waiting for fetal asystole was particularly difficult. - Despite being challenging, providers believed that feticide was an important task and helped to reduce trauma for the parents by ensuring there were no signs of life. | 3 |
| [87] | Harries, 2009 | South Africa  38 providers  Doctor, Nurse/Midwife, RN, Enrolled Nurse, Counsellor, Management | Comprehensive abortion service | Qualitative  Interviews, Focus groups  Thematic analysis | - High levels of conscientious objection created issues in providing access to care, though the new law “empowered” mid-level providers. - Many discussed moral reasons for providing care, such as preventing maternal mortality, as well as their professional duty to care. - Providers supported abortion in cases of rape or incest and for lower socio-economic groups, though had greater issues with second trimester and repeat abortions and refusal of contraception. - They felt that the services were under-resourced. | 4 |
| [88] | Lipp, 2008 | United Kingdom  12 providers  Nurses, Midwives | Comprehensive abortion service | Qualitative  Interviews  Grounded theory | - Nurses and midwives in the UK said that it was important to accept their patient’s decision to access abortion and to respect the specific circumstances and have preceded that decision. - They believed “going through the motions” of medical abortion would help to reduce unwanted repeat abortions. - They also emphasised the importance of providing a woman-centred service. | 4 |

| **Ref** | **Author, Year** | **Country**  **Sample Size**  **Professions** | **Procedures Provided ^a^** | **Methodology**  **Data Collection Method**  **Analysis** | **Key Findings** | **MMAT Score ^b^** |
| --- | --- | --- | --- | --- | --- | --- |
| [89] | Ordinioha, 2008 | Nigeria  34 providers  Doctors | Early abortion (illegally) | Mixed methods  Interviews, survey  Statistical analyses, qual analyses not described | - In response to legal restrictions on abortion care, providers could only provide care in select cases. - Some had developed further protections, such as encrypted forms. - The minority had experienced problems with law enforcement. | 0 |
| [90] | Garel, 2007 | France  87 providers  Midwives | Medical abortion for fatal fetal abnormalities | Mixed methods  Survey  Statistical analyses & content analyses | - Midwives believed that aspects of care were difficult, particularly emotional support, feelings of loneliness and caring for the fetus. - Most experienced emotional difficulties when providing care. - They wanted greater training in the legislation and in how to support women. - Despite difficulties, they believed abortion was part of their work. | 2 |
| [91] | Lindström, 2007 | Sweden  139 providers  Midwives | Comprehensive abortion service | Quantitative (Cross-sectional observational)  Survey  Statistical analyses | - Misgivings about abortion care were more common among midwives who were not regularly involved in care. - Most supported the services, with two-thirds believing that working with abortion had brought them good experiences. | 2 |
| [92] | Wolkomir, 2007 | United States  9 providers  Counsellors, Managers, Back Staffers, Receptionist | Comprehensive abortion service | Qualitative  Interviews  Grounded theory | - Most providers came to their work with a desire to support and help women. - They found comfort in investing in patients they saw as easy, i.e. those who were comfortable in their decision to end their pregnancy. - They emotionally detached from hostile patients and those who were unsure of their decision. - In difficult cases, they had to build boundaries to adequately support their patient without facing personal emotional trauma. | 3 |
| [93] | Hammarstedt, 2006 | Sweden  224 providers  Gynaecologists | Comprehensive abortion service | Mixed methods  Survey  Statistical analyses | - A quarter of obstetricians held misgivings about abortion, with half holding misgivings about later-gestation care. - A minority had considered leaving their job. - More than half felt that providing care brought them good experiences. | 2 |

| **Ref** | **Author, Year** | **Country**  **Sample Size**  **Professions** | **Procedures Provided ^a^** | **Methodology**  **Data Collection Method**  **Analysis** | **Key Findings** | **MMAT Score ^b^** |
| --- | --- | --- | --- | --- | --- | --- |
| [94] | Mokgethi, 2006 | South Africa  25 providers  Nurses | Comprehensive abortion service | Quantitative (Cross-sectional observational)  Survey  Statistical analyses | - Most nurses received support from other colleagues working in abortion care. A minority received support from other nurses not working in the abortion services, and less from their family. - Some perceived management as unsupportive. | 4 |
| [95] | Statham, 2006 | United Kingdom  15 providers  Doctors, Midwives | Abortion for fatal fetal abnormalities | Qualitative  Interviews  Unnamed analysis described | - Fetal medicine specialists discussed the difficulties of ensuring that they worked within the law and within their own ethical frameworks when making decisions about offering terminations after viability. - They talked about the importance of discussing concerns with colleagues. | 3 |
| [96] | Chiappetta-Swanson, 2005 | Canada  41 providers  Nurses, Nursing Directors, Clinical Nurse Specialists | Abortion for fatal fetal abnormalities | Qualitative  Interviews  Grounded theory | - Nurses believed that abortion should be provided in labour and delivery units and that they were unable to provide best care to patients. - They also noted a lack of professional back-up when providing, including doctors, social workers, and chaplains. - There was also a lack of policies from the hospital, and many said that they didn’t have a choice about becoming a provider. - They also discussed the high levels of physical and emotional care involved in care and how, in the absence of support, they had to support each other. | 5 |
| [97] | Hanna, 2005 | United States  10 providers  Nurses | Comprehensive abortion service | Qualitative  Interviews  Unnamed analysis described | - There were five properties of the lived experience of moral distress among the nurses: perception, pain, valuing, altered participation, and perspective. - Moral distress was described as either shock, muted, or suppressed. - Type of moral distress was related to situational conditions, recognition of moral ends, quality of coping processes, and temporal breadth | 5 |
| [98] | Mayers, 2005 | South Africa  3 providers  Nurses, Midwives | Comprehensive abortion service | Qualitative  Interviews  Hycner’s guidelines | - Providers experienced obstacles with some patients, such as those who refused contraception, showed no emotion, or were rude. - They experienced mostly negative emotions when assisting with the abortion care procedure, including guilt, anger, hopeless, and isolated. - They experienced moral/ethical conflicts during their work, particularly when confronted by the fetus or when caring for teenagers. - Providers sought support from patients, staff, and family members, though believed that managers were unsupportive. | 3 |

| **Ref** | **Author, Year** | **Country**  **Sample Size**  **Professions** | **Procedures Provided ^a^** | **Methodology**  **Data Collection Method**  **Analysis** | **Key Findings** | **MMAT Score ^b^** |
| --- | --- | --- | --- | --- | --- | --- |
| [99] | Potgrier, 2004 | South Africa  22 providers  Nurses | Comprehensive abortion service | Qualitative  Interviews, Focus Groups,  Grounded theory | - Many chose to become a provider to lower unsafe abortion and emphasised the social importance of a safe abortion service and access to abortion as a fundamental human right. Many also believed that traditional forms of abortion have been in South African culture for a long time. - Many religious providers believed that God would want them to provide care, and providers separated themselves from anti-choice rhetoric. | 3 |
| [100] | da Costa, 2003 | South Africa  15 providers  Obstetricians | Comprehensive abortion service | Qualitative  Interviews  Grounded theory | - Obstetricians in South Africa said that their views about abortion were informed by internal role forces, such as personal support about abortion, believing that life begins at conception, religious beliefs, and duty of care. - External role forces were also prominent. These included the expectation to provide, high workload, and pressure to provide from the legislation to secure promotions, and from patients. | 1 |
| [101] | Cignacco, 2002 | Switzerland  13 providers  Midwives | Medical abortion for fatal fetal abnormalities | Qualitative  Interviews  Content analysis | - Midwives experienced a conflict between their duty to provide abortion and preserve life and sadness that abortion resulted in the end of potential life, something that they tried to “blot out”. - Despite this, all supported women’s right to self-determination and believed that abortion was within their scope of practice. - Midwives wanted greater involved in the decision-making process. | 4 |
| [102] | Garel, 2002 | France  47 providers  Obstetricians, Midwives | Abortion for fatal fetal abnormalities | Qualitative  Interviews  Analysis not described | - Most providers struggled with abortion for non-fatal abnormalities, which raised an issue about the place of disabilities in society. - Providers talked positively about the advent of feticide, as it ensured fetal signs of life were not present at delivery as had previously been - Providers believed it was important to discuss the challenging aspects of their work with their colleagues. | 2 |
| [103] | Askey, 2001 | United Kingdom  23 providers  Obstetricians, Link worker, Ultrasonographers, Nurses, Midwives | Abortion for fatal fetal abnormalities | Qualitative  Interviews  Grounded theory | - The providers ability to handle the challenges of later-gestation abortion matured with increased clinical and personal experience. - When first starting, providers have little experience and are uncertain about how to appropriately care for patients. - With experience, providers learn to set boundaries, develop empathy, and acknowledge their own limits. - When they have amassed enough experience, they are knowledgeable and confident in their ability to provide care, as well as to distance themselves from the situation to avoid becoming upset. | 2 |
| **Ref** | **Author, Year** | **Country**  **Sample Size**  **Professions** | **Procedures Provided ^a^** | **Methodology**  **Data Collection Method**  **Analysis** | **Key Findings** | **MMAT Score ^b^** |
| [104] | Gmeimer, 2000 | South Africa  Not mentioned  Nurses | Comprehensive abortion service | Qualitative  Interviews  Tesch’s method of qualitative analysis | - Nurses in South Africa experienced stigma related to their abortion work, with many with-holding disclosure to avoid stigma. - Some experienced negative emotions such as value conflict, emotional fatigue, becoming moralistic, and judging patients. - Some also experienced positive emotions such as empathy and respect. - Nurses called for more cognitive, emotional, and spiritual supports. | 2 |
| [105] | Fitzpatrick, 1999 | United States  71 providers  Security Personnel, Escorts, Directors, Administrators, Receptionists, Counsellors, Physicians, Nurses, Lab Technicians, Medical Assistants | Comprehensive abortion service | Mixed Methods  Interviews, Survey  Statistical analyses | - Providers in the Southeast of the United States experienced stigma related to their abortion work. - Many experienced moderate forms of violence against them and many witnessed a greater variety and number of violent acts. - Many reported symptoms of post-traumatic stress disorder, which were related to witnessing violent acts. | 0 |
| [106] | Donnay, 1993 | Belgium  143 providers  Obstetricians, GPs, Psychologists, Nurses, Social Workers | Comprehensive abortion service | Quantitative (Cross-sectional observational)  Survey  Statistical analyses | - Providers in Belgium were increasingly motivated to provide care to alleviate women’s problems. - Many had negative emotional reactions to repeat abortions and later-gestation abortions. - Many had faced judicial proceedings against their practice of care. | 2 |
| ^a^ “Comprehensive abortion service” equates to the legal provision of abortion in that country. See S4 Table for an overview of the abortion services for each country included in this review.  ^B^ Scores according to the authors rating using the Mixed Methods Appraisal Tool (MMAT). See S3 Table for a detailed appraisal of the included studies. | | | | | | |

**References**

1. Chowdhary P, Newton-Levinson A, Rochat R. "No one does this for the money or lifestyle": Abortion providers' perspectives on factors affecting workforce recruitment and retention in the southern United States. Matern Child Health J. 2022;26(6):1350-7.

2. Mainey L, O'Mullan C, Reid-Searl K. Working with or against the system: Nurses' and midwives' process of providing abortion care in the context of gender-based violence in Australia. J Adv Nurs 2022 Epub 2022 March 14 Available from: 101111/jan15226.

3. Newton-Levinson A, Higdon M, Rochat R. Supporting staff in southern family planning clinics: Challenges and opportunities. Matern Child Health J. 2022;26(2):319-27.

4. Reeves JA, Goedken P, Hall KS, Lee SC, Cwiak CA. Anesthesia providers' perspectives on abortion provision: Deductive findings from a qualitative study. Int J Obstet Anesth. 2022;49:103239.

5. Armour S, Gilkison A, Hunter M. Midwives holding the space for women undergoing termination of pregnancy: A qualitative inquiry. Women Birth. 2021;34(6):e616-23.

6. Becker A, Hann LR. "It makes it more real": Examining ambiguous fetal meanings in abortion care. Soc Sci Med. 2021;272:113736.

7. Cannon R, White K, Seifert B, Woodhams E, Brandi K, Yinusa-Nyahkoon L. Exploring the physician's role in contraceptive counseling at the time of abortion in the US. Contraception. 2021;103(5):316-21.

8. Dempsey B, Favier M, Mullally A, Higgins MF. Exploring providers' experience of stigma following the introduction of more liberal abortion care in the Republic of Ireland. Contraception. 2021;104(4):414-9.

9. Ewnetu DB, Thorsen VC, Solbakk JH, Magelssen M. Navigating abortion law dilemmas: Experiences and attitudes among Ethiopian health care professionals. BMC Med Ethics. 2021;22(1):166.

10. Magelssen M, Ewnetu DB. Professionals' experience with conscientious objection to abortion in Addis Ababa, Ethiopia: An interview study. Dev World Bioeth. 2021;21(2):68-73.

11. McLeod C, Javlekar A, Flink-Bochacki R. Exploring the relationship between abortion provision and providers' personal pregnancy and parenting experiences. WHI. 2021;31(2):171-6.

12. Persson M, Larsson EC, Islam NP, Gemzell-Danielsson K, Klingberg-Allvin M. A qualitative study on health care providers' experiences of providing comprehensive abortion care in Cox's Bazar, Bangladesh. Confl Health. 2021;15(1):6.

13. Rasmussen KN, Janiak E, Cottrill AA, Stulberg DB. Expanding access to medication abortion through pharmacy dispensing of mifepristone: Primary care perspectives from Illinois. Contraception. 2021;104(1):98-103.

14. Simmonds K, Schwartz-Barcott D, Erickson-Owens D. Nurse practitioners' and certified nurse midwives' experiences providing comprehensive early abortion care in New England, USA. Health Care Women Int. 2021;17:1-23.

15. Zwerling B, Rousseau J, Ward KM, Olshansky E, Lo A, Thiel de Bocanegra H, et al. "It's a horrible assignment": A qualitative study of labor and delivery nurses' experience caring for patients undergoing labor induction for fetal anomalies or fetal demise. Contraception. 2021;104(3):301-4.

16. Aborigo RA, Moyer CA, Sekwo E, Kuwolamo I, Kumaga E, Oduro AR, et al. Optimizing task-sharing in abortion care in Ghana: Stakeholder perspectives. Int J Gynecol Obstet. 2020;150(S1):17-24.

17. Ewnetu DB, Thorsen VC, Solbakk JH, Magelssen M. Still a moral dilemma: How Ethiopian professionals providing abortion come to terms with conflicting norms and demands. BMC Med Ethics. 2020;21(1):16.

18. Hasselbacher LA, Hebert LE, Liu Y, Stulberg DB. "My hands are tied": Abortion restrictions and providers' experiences in religious and nonreligious health care systems. Perspect Sex Reprod Health. 2020;52(2):107-15.

19. Mavuso JMJ, Macleod CI. Resisting abortion stigma in situ: South African womxn's and healthcare providers' accounts of the pre-abortion counselling healthcare encounter. Cult Health Sex. 2020;22(11):1299-313.

20. Maxwell KJ, Hoggart L, Bloomer F, Rowlands S, Purcell C. Normalising abortion: What role can health professionals play? BMJ Sex Reprod Health. 2020;47:32-6.

21. Mosley EA, Martin L, Seewald M, Hassinger J, Blanchard K, Baum SE, et al. Addressing abortion provider stigma: A pilot implementation of the Providers Share Workshop in Sub-Saharan Africa and Latin America. Int Perspect Sex Reprod Health. 2020;46:35-50.

22. Nkosi LJ, Mulaudzi FM, Peu MD. Challenges related to the structure of the choice on termination of pregnancy services in public health facilities in the Tshwane District of Gauteng. Afr J Reprod Health. 2020;24(1):106-14.

23. Påfs J, Rulisa S, Klingberg-Allvin M, Binder-Finnema P, Musafili A, Essén B. Implementing the liberalized abortion law in Kigali, Rwanda: Ambiguities of rights and responsibilities among health care providers. Midwifery. 2020;80:102568.

24. Power S, Meaney S, O'Donoghue K. Fetal Medicine Specialists' experiences of providing a new service of termination of pregnancy for fatal fetal anomaly: A qualitative study. BJOG. 2020;128(4):676-84.

25. Ramón Michel A, Kung S, López-Salm A, Ariza Navarrete S. Regulating conscientious objection to legal abortion in Argentina - Taking into consideration its uses and consequences. Health Hum Rights. 2020;22(2):271-83.

26. Teffo M, Rispel L. Resilience or detachment? Coping strategies among termination of pregnancy health care providers in two South African provinces. Cult Health Sex. 2020;22(3):336-51.

27. Zaręba K, Banasiewicz J, Rozenek H, Ciebiera M, Jakiel G. Emotional complications in midwives participating in pregnancy termination procedure: Polish experience. Int J Environ Res Public Health. 2020;17(8).

28. Czarnecki D, Anspach RR, De Vries RG, Dunn MD, Hauschildt K, Harris LH. Conscience reconsidered: The moral work of navigating participation in abortion care on labor and delivery. Soc Sci Med. 2019;232:181-9.

29. Fernández Vázquez SS, Brown J. From stigma to pride: Health professionals and abortion policies in the Metropolitan Area of Buenos Aires. Sex Reprod Health Matters. 2019;27(3):1691898.

30. McLean E, Desalegn DN, Blystad A, Miljeteig I. When the law makes doors slightly open: Ethical dilemmas among abortion service providers in Addis Ababa, Ethiopia. BMC Med Ethics. 2019;20(1):60.

31. Oelhafen S, Monteverde S, Cignacco E. Exploring moral problems and moral competences in midwifery: A qualitative study. Nurs Ethics. 2019;26(5):1373-86.

32. Seewald M, Martin LA, Echeverri L, Njunguru J, Hassinger JA, Harris LH. Stigma and abortion complications: Stories from three continents. Sex Reprod Health Matters. 2019;27(3):1688917.

33. Cárdenas R, Labandera A, Baum SE, Chiribao F, Leus I, Avondet S, et al. "It's something that marks you": Abortion stigma after decriminalization in Uruguay. Reprod Health. 2018;15(1):150.

34. De Zordo S. From women's 'irresponsibility' to foetal 'patienthood': Obstetricians-gynaecologists' perspectives on abortion and its stigmatisation in Italy and Cataluña. Glob Public Health. 2018;13(6):711-23.

35. Greenberg S, Nothnagle M. An "invaluable skill": Reflections on abortion training and postresidency practice. Fam Med. 2018;50(9):691-3.

36. Janiak E, Freeman S, Maurer R, Berkman LF, Goldberg AB, Bartz D. Relationship of job role and clinic type to perceived stigma and occupational stress among abortion workers. Contraception. 2018;98(6):517-21.

37. Martin LA, Hassinger JA, Seewald M, Harris LH. Evaluation of abortion stigma in the workforce: Development of the Rrevised Abortion Providers Stigma Scale. WHI. 2018;28(1):59-67.

38. Puri MC, Raifman S, Khanal B, Maharjan DC, Foster DG. Providers' perspectives on denial of abortion care in Nepal: A cross sectional study. Reprod Health. 2018;15(1):170.

39. Britton LE, Mercier RJ, Buchbinder M, Bryant AG. Abortion providers, professional identity, and restrictive laws: A qualitative study. Health Care Women Int. 2017;38(3):222-37.

40. Dawson AJ, Nicolls R, Bateson D, Doab A, Estoesta J, Brassil A, et al. Medical termination of pregnancy in general practice in Australia: A descriptive-interpretive qualitative study. Reprod Health. 2017;14(1):39.

41. Martin LA, Hassinger JA, Debbink M, Harris LH. Dangertalk: Voices of abortion providers. Soc Sci Med. 2017;184:75-83.

42. Mauri PA, Squillace F. The experience of Italian nurses and midwives in the termination of pregnancy: A qualitative study. Eur J Contracept Reprod Health Care. 2017;22(3):227-32.

43. Purcell C, Cameron S, Lawton J, Glasier A, Harden J. The changing body work of abortion: A qualitative study of the experiences of health professionals. Sociol Health Illn. 2017;39(1):78-94.

44. Teffo ME, Rispel LC. 'I am all alone': Factors influencing the provision of termination of pregnancy services in two South African provinces. Glob Health Action. 2017;10(1):1347369.

45. Aniteye P, O'Brien B, Mayhew SH. Stigmatized by association: Challenges for abortion service providers in Ghana. BMC Health Serv Res. 2016;16(1):486.

46. Debbink MLP, Hassinger JA, Martin LA, Maniere E, Youatt E, Harris LH. Experiences with the Providers Share Workshop method: Abortion worker support and research in tandem. Qual Health Res. 2016;26(13):1823-37.

47. Fay V, Thomas S, Slade P. Maternal-fetal medicine specialists' experiences of conducting feticide as part of termination of pregnancy: A qualitative study. Prenat Diagn. 2016;36(1):92-9.

48. Larsson EC, Fried S, Essén B, Klingberg-Allvin M. Equitable abortion care – A challenge for health care providers. Experiences from abortion care encounters with immigrant women in Stockholm, Sweden. Sex Reprod Healthc. 2016;10:14-8.

49. Purcell C, Cameron S, Lawton J, Glasier A, Harden J. Contraceptive care at the time of medical abortion: Experiences of women and health professionals in a hospital or community sexual and reproductive health context. Contraception. 2016;93(2):170-7.

50. Yang CF, Che HL, Hsieh HW, Wu SM. Concealing emotions: Nurses' experiences with induced abortion care. J Clin Nurs. 2016;25(9-10):1444-54.

51. Black KI, Douglas H, de Costa C. Women's access to abortion after 20 weeks' gestation for fetal chromosomal abnormalities: Views and experiences of doctors in New South Wales and Queensland. Aust N Z J Obstet Gynaecol. 2015;55(2):144-8.

52. Mauri PA, Ceriotti E, Soldi M, Contini NNG. Italian midwives' experiences of late termination of pregnancy. A phenomenological–hermeneutic study. Nurs Health Sci. 2015;17(2):243-9.

53. McLemore MR, Kools S, Levi AJ. Calculus formation: Nurses’ decision‐making in abortion‐related care. Res Nursing Health. 2015;38(3):222-31.

54. McLemore MR, Levi A, Angel James E. Recruitment and retention strategies for expert nurses in abortion care provision. Contraception. 2015;91(6):474-9.

55. Mercier RJ, Buchbinder M, Bryant A, Britton L. The experiences and adaptations of abortion providers practicing under a new TRAP law: A qualitative study. Contraception. 2015;91(6):507-12.

56. Potdar P, Barua A, Dalvie S, Pawar A. "If a woman has even one daughter, I refuse to perform the abortion": Sex determination and safe abortion in India. Reprod Health Matters. 2015;23(45):114-25.

57. Strefling IdSS, Lunardi Filho WD, Kerber NPdC, Soares MC, Ribeiro JP. Nursing perceptions about abortion management and care: A qualitative study. Texto Contexto Enferm. 2015;24(3):784-91.

58. Andersson IM, Gemzell-Danielsson K, Christensson K. Caring for women undergoing second-trimester medical termination of pregnancy. Contraception. 2014;89(5):460-5.

59. Gwangwa TJ, Kgole JC, Matlala F. Experiences of registered midwives performing termination of pregnancy at Polokwane Mankweng Hospital Complex, Limpopo Province, South Africa. Afr J Phys Health Educ Recreat Dance. 2014;1(2):261-74.

60. Martin LA, Debbink M, Hassinger J, Youatt E, Eagen-Torkko M, Harris LH. Measuring stigma among abortion providers: Assessing the Abortion Provider Stigma Survey. Women Health. 2014;54(7):641-61.

61. Martin LA, Debbink M, Hassinger J, Youatt E, Harris LH. Abortion providers, stigma and professional quality of life. Contraception. 2014;90(6):581-7.

62. Parker A, Swanson H, Frunchak V. Needs of labor and delivery nurses caring for women undergoing pregnancy termination. J Obstet Gynecol Neonatal Nurs. 2014;43(4):478-87.

63. Aniteye P, Mayhew SH. Shaping legal abortion provision in Ghana: Using policy theory to understand provider-related obstacles to policy implementation. Health Res Policy Syst. 2013;11:23.

64. Christensen AV, Christiansen AH, Petersson B. Faced with a dilemma: Danish midwives' experiences with and attitudes towards late termination of pregnancy. Scand J Caring Sci. 2013;27(4):913-20.

65. Dressler J, Maughn N, Soon JA, Norman WV. The perspective of rural physicians providing abortion in Canada: qualitative findings of the British Columbia Abortion Providers Survey (BCAPS). PLoS One. 2013;8(6):e67070.

66. Mizuno M, Kinefuchi E, Kimura R, Tsuda A. Professional quality of life of Japanese nurses/midwives providing abortion/childbirth care. Nurs Ethics. 2013;20(5):539-50.

67. Norman WV, Soon JA, Maughn N, Dressler J. Barriers to rural induced abortion services in Canada: findings of the British Columbia Abortion Providers Survey (BCAPS). PLoS One. 2013;8(6):e67023.

68. Turk JK, Steinauer JE, Landy U, Kerns JL. Barriers to D&E practice among family planning subspecialists. Contraception. 2013;88(4):561-7.

69. Harries J, Lince N, Constant D, Hargey A, Grossman D. The challenges of offering public second trimester abortion services in South Africa: Health care providers' perspectives. J Biosoc Sci. 2012;44(2):197-208.

70. Möller A, Öfverstedt S, Siwe K. Proud, not yet satisfied: The experiences of abortion service providers in the Kathmandu Valley, Nepal. Sex Reprod Healthc. 2012;3(4):135-40.

71. Perrin E, Berthoud M, Pott M, Vera AGT, Bianchi-Demicheli F. Views of healthcare professionals dealing with legal termination of pregnancy up to 12 WA in French-speaking Switzerland. Swiss Med Wkly. 2012;142:w13584.

72. Puri M, Lamichhane P, Harken T, Blum M, Harper CC, Darney PD, et al. "Sometimes they used to whisper in our ears": Health care workers' perceptions of the effects of abortion legalization in Nepal. BMC Public Health. 2012;12.

73. Contreras X, van Dijk MG, Sanchez T, Smith PS. Experiences and opinions of health‐care professionals regarding legal abortion in Mexico City: A qualitative study. Stud Fam Plan. 2011;42(3):183-90.

74. Halldén BM, Lundgren I, Christensson K. Ten Swedish midwives' lived experiences of the care of teenagers' early induced abortions. Health Care Women Int. 2011;32(5):420-40.

75. Harris LH, Debbink M, Martin L, Hassinger J. Dynamics of stigma in abortion work: Findings from a pilot study of the Providers Share Workshop. Soc Sci Med. 2011;73(7):1062-70.

76. Lamichhane P, Harken T, Puri M, Darney PD, Blum M, Harper CC, et al. Sex-selective abortion in Nepal: A qualitative study of health workers' perspectives. WHI. 2011;21(3):S37-S41.

77. Lindström M, Wulff M, Dahlgren L, Lalos A. Experiences of working with induced abortion: Focus group discussions with gynaecologists and midwives/nurses. Scand J Caring Sci. 2011;25(3):542-8.

78. Lipp A. Self-preservation in abortion care: A grounded theory study. J Clin Nurs. 2011;20(5-6):892-900.

79. Mizuno M. Confusion and ethical issues surrounding the role of Japanese midwives in childbirth and abortion: A qualitative study. Nurs Health Sci. 2011;13(4):502-6.

80. O'Donnell J, Weitz TA, Freedman LR. Resistance and vulnerability to stigmatization in abortion work. Soc Sci Med. 2011;73(9):1357-64.

81. Freedman L, Landy U, Darney P, Steinauer J. Obstacles to the integration of abortion into obstetrics and gynecology practice. Perspect Sex Reprod Health. 2010;42(3):146-51.

82. Gallagher K, Porock D, Edgley A. The concept of 'nursing' in the abortion services. J Adv Nurs. 2010;66(4):849-57.

83. Lipp A. Conceding and concealing judgement in termination of pregnancy: A grounded theory study. J Res Nurs. 2010;15(4):365-78.

84. Mamabolo LRC, Tjallinks JE. Experiences of registered nurses at one community health centre near Pretoria providing termination of pregnancy services. Afr J Nurs Midwifery. 2010;12(1):73-86.

85. Nicholson J, Slade P, Fletcher J. Termination of pregnancy services: Experiences of gynaecological nurses. J Adv Nurs. 2010;66(10):2245-56.

86. Graham RH, Mason K, Rankin J, Robson SC. The role of feticide in the context of late termination of pregnancy: A qualitative study of health professionals' and parents' views. Prenat Diagn. 2009;29(9):875-81.

87. Harries J, Stinson K, Orner P. Health care providers' attitudes towards termination of pregnancy: A qualitative study in South Africa. BMC Public Health. 2009;9:296.

88. Lipp A. A woman centred service in termination of pregnancy: A grounded theory study. Contemp Nurse. 2008;31(1):9-19.

89. Ordinioha B, Brisibe S. Clandestine abortion in Port Harcourt: providers' motivations and experiences. Niger J Med. 2008;17(3):291-5.

90. Garel M, Etienne E, Blondel B, Dommergues M. French midwives' practice of termination of pregnancy for fetal abnormality. At what psychological and ethical cost? Prenat Diagn. 2007;27(7):622-8.

91. Lindström M, Jacobsson L, Wulff M, Lalos A. Midwives' experiences of encountering women seeking an abortion. J Psychosom Obstet Gynaecol. 2007;28(4):231-7.

92. Wolkomir M, Powers J. Helping women and protecting the self: The challenge of emotional labor in an abortion clinic. Qual Sociol. 2007;30(2):153-69.

93. Hammarstedt M, Lalos A, Wulff M. A population-based study of Swedish gynecologists' experiences of working in abortion care. Acta Obstet Gynecol Scand. 2006;85(2):229-35.

94. Mokgethi NE, Ehlers VJ, van der Merwe MM. Professional nurses' attitudes towards providing termination of pregnancy services in a tertiary hospital in the north west province of South Africa. Curationis. 2006;29(1):32-9.

95. Statham H, Solomou W, Green J. Late termination of pregnancy: Law, policy and decision making in four English fetal medicine units. BJOG. 2006;113(12):1402-11.

96. Chiappetta-Swanson C. Dignity and dirty work: Nurses' experiences in managing genetic termination for fetal anomaly. Qual Sociol. 2005;28(1):93-116.

97. Hanna DR. The lived experience of moral distress: Nurses who assisted with elective abortions. Res Theory Nurs Pract. 2005;19(1):95-124.

98. Mayers PM, Parkes B, Green B, Turner J. Experiences of registered midwives assisting with termination of pregnancies at a tertiary level hospital. Health SA Gesondheid. 2005;10(1):15-25.

99. Potgrier C, Andrews G. South African nurses' accounts for choosing to be termination of pregnancy providers. Health SA Gesondheid. 2004;9(2):20-30.

100. da Costa PC, Donald F. The experience of person-role conflict in doctors expected to terminate pregnancies in the South African public sector. S Afr J Psychol. 2003;33(1):10-8.

101. Cignacco E. Between professional duty and ethical confusion: Midwives and selective termination of pregnancy. Nurs Ethics. 2002;9(2):179-91; discussion 91-3.

102. Garel M, Gosme-Seguret S, Kaminski M, Cuttini M. Ethical decision-making in prenatal diagnosis and termination of pregnancy: A qualitative survey among physicians and midwives. Prenat Diagn. 2002;22(9):811-7.

103. Askey K, Moss L. Termination for fetal defects: The effect on midwifery staff. Br J Midwifery. 2001;9(1):17-24.

104. Gmeiner AC, Van Wyk S, Poggenpoel M, Myburgh CP. Support for nurses directly involved with women who chose to terminate a pregnancy. Curationis. 2000;23(1):70-8.

105. Fitzpatrick KM, Wilson M. Exposure to violence and posttraumatic stress symptomatology among abortion clinic workers. J Trauma Stress. 1999;12(2):227-42.

106. Donnay F, Bregentzer A, Leemans P, Verougstraete A, Vekemans M. Safe abortions in an illegal context: Perceptions from service providers in Belgium. Stud Fam Plann. 1993;24(3):150-62.
