# Supplementary material for: Providers’ experiences with abortion care: A scoping review
Source: PLoS One. 2024 Jul 1;19(7):e0303601. doi: 10.1371/journal.pone.0303601 (PMC11216598; doi:10.1371/journal.pone.0303601)
Supplement: S3 Table — (DOCX) [file pone.0303601.s003.docx]

**S3 Table**

**Overview of the quality appraisal on the studies included in the scoping review on providers’ experiences with abortion care**

**Overview of the Quality Appraisal**

Using the MMAT, all studies were appraised for methodological quality. In general, the studies showed high methodological quality, with 34 studies (32.1%) meeting all five respective questions and 23 (21.7%) meeting at least four. Twenty studies (18.9%) met good methodological quality with three criteria, while 19 studies (17.9%) met fair methodological quality with two criteria. Ten studies were deemed poor methodological quality as they only met one criterion (*n* = 8; 7.5%) or none (*n* = 2; 1.9%).

A detailed description for each study can be found in Tables 4a, 4b, 4c, and 4d. In brief, each study type had their own issues regarding methodological quality. None of the nine quantitative non-randomised studies included a representative sample. This was also an issue for the quantitative descriptive studies, with only one of the six studies meeting this criterion. The measures and analyses were generally appropriate to meet this criterion, with some studies failing to control for potential confounders in the analyses. The appraisal of the qualitative studies was more nuanced. Most qualitative studies sufficiently supported their findings with quotes and many collected data using adequate methods. A minority (*n* = 4, 4.8%) did not include quotes or used them too sparingly. More studies (*n* = 25, 30.1%) failed to adequately describe their data collection methods, particularly giving a lack of detail on their sample size and composition and what was discussed during the interview. Twenty-one studies (25.3%) failed to give enough information on the guiding qualitative approach. Thirty-one studies (37.3%) failed to provide adequate information on their analyses. Of these, 18 did not give enough indication of the steps they took, while the remaining 13 described steps that were inappropriate use of the guiding methodology. Thirty-five studies (42.2%) did not meet the final MMAT criterion (clear links between data collection, analyses, and findings), particularly given a lack of detail about the analyses or the sample making it difficult to draw links between the methods. Finally, most of the mixed methods studies were of low-methodological quality. Only one of the eight studies satisfied all five criteria, with another satisfying four. Seven studies did not include a justification for the use of quantitative and qualitative data, six did not give enough information on how they integrated the qualitative and quantitative research findings, and five studies did not adhere to quality standards for the individual components.

| **S3 Table 1:** Qualitative Study Design | | | | | | |
| --- | --- | --- | --- | --- | --- | --- |
| **Ref** | **Author, Year** | **1.1 Is the qualitative approach appropriate to answer the research questions?** | **1.2 Are the qualitative data collection methods adequate to address the research questions?** | **1.3 Are the findings adequately derived from the data?** | **1.4 Is the interpretation of results sufficiently substantiated by data?** | **1.5 Is there coherence between qualitative data sources, collection, analysis, and interpretation?** |
| [1] | Chowdhary, 2022 |  |  |  |  |  |
| [2] | Mainey, 2022 |  |  |  |  |  |
| [3] | Newtown-Levinson, 2022 |  |  |  |  |  |
| [4] | Reeves, 2022 |  |  |  |  |  |
| [5] | Armour, 2021 |  |  |  |  |  |
| [6] | Becker, 2021 |  |  |  |  |  |
| [7] | Cannon, 2021 |  |  |  |  |  |
| [8] | Ewnetu, 2021 |  |  |  |  |  |
| [9] | Magelssen, 2021 |  |  |  |  |  |
| [10] | McLeod, 2021 |  |  |  |  |  |
| [11] | Persson, 2021 |  |  |  |  |  |
| [12] | Rasmussen, 2021 |  |  |  |  |  |
| [13] | Simmonds, 2021 |  |  |  |  |  |
| [14] | Zwerling, 2021 |  |  |  |  |  |
| [15] | Aborigo, 2020 |  |  |  |  |  |
| [16] | Ewnetu, 2020 |  |  |  |  |  |
| [17] | Hasselbacher, 2020 |  |  |  |  |  |
| **Ref** | **Author, Year** | **1.1 Is the qualitative approach appropriate to answer the research questions?** | **1.2 Are the qualitative data collection methods adequate to address the research questions?** | **1.3 Are the findings adequately derived from the data?** | **1.4 Is the interpretation of results sufficiently substantiated by data?** | **1.5 Is there coherence between qualitative data sources, collection, analysis, and interpretation?** |
| [18] | Mavuso, 2020 |  |  |  |  |  |
| [19] | Maxwell, 2020 |  |  |  |  |  |
| [20] | Påfs, 2020 |  |  |  |  |  |
| [21] | Power, 2020 |  |  |  |  |  |
| [22] | Teffo, 2020 |  |  |  |  |  |
| [23] | Czarnecki, 2019 |  |  |  |  |  |
| [24] | Fernández, 2019 |  |  |  |  |  |
| [25] | McLean, 2019 |  |  |  |  |  |
| [26] | Oelhafen, 2019 |  |  |  |  |  |
| [27] | Seewald, 2019 |  |  |  |  |  |
| [28] | Cárdenas, 2018 |  |  |  |  |  |
| [29] | Greenberg, 2018 |  |  |  |  |  |
| [30] | Britton, 2017 |  |  |  |  |  |
| [31] | Dawson, 2017 |  |  |  |  |  |
| [32] | Martin, 2017 |  |  |  |  |  |
| [33] | Mauri, 2017 |  |  |  |  |  |
| [34] | Purcell, 2017 |  |  |  |  |  |
| [35] | Aniteye, 2016 |  |  |  |  |  |
| **Ref** | **Author, Year** | **1.1 Is the qualitative approach appropriate to answer the research questions?** | **1.2 Are the qualitative data collection methods adequate to address the research questions?** | **1.3 Are the findings adequately derived from the data?** | **1.4 Is the interpretation of results sufficiently substantiated by data?** | **1.5 Is there coherence between qualitative data sources, collection, analysis, and interpretation?** |
| [36] | Debbink, 2016 |  |  |  |  |  |
| [37] | Fay, 2016 |  |  |  |  |  |
| [38] | Larsson, 2016 |  |  |  |  |  |
| [39] | Purcell, 2016 |  |  |  |  |  |
| [40] | Yang, 2016 |  |  |  |  |  |
| [41] | Black, 2015 |  |  |  |  |  |
| [42] | Mauri, 2015 |  |  |  |  |  |
| [43] | McLemore, 2015a |  |  |  |  |  |
| [44] | McLemore, 2015b |  |  |  |  |  |
| [45] | Mercier, 2015 |  |  |  |  |  |
| [46] | Potdar, 2015 |  |  |  |  |  |
| [47] | Strefling, 2015 |  |  |  |  |  |
| [48] | Andersson, 2014 |  |  |  |  |  |
| [49] | Gwangwa, 2014 |  |  |  |  |  |
| [50] | Parker, 2014 |  |  |  |  |  |
| [51] | Aniteye, 2013 |  |  |  |  |  |
| [52] | Christensen, 2013 |  |  |  |  |  |
| [53] | Dressler, 2013 |  |  |  |  |  |
| **Ref** | **Author, Year** | **1.1 Is the qualitative approach appropriate to answer the research questions?** | **1.2 Are the qualitative data collection methods adequate to address the research questions?** | **1.3 Are the findings adequately derived from the data?** | **1.4 Is the interpretation of results sufficiently substantiated by data?** | **1.5 Is there coherence between qualitative data sources, collection, analysis, and interpretation?** |
| [54] | Harries, 2012 |  |  |  |  |  |
| [55] | Möller, 2012 |  |  |  |  |  |
| [56] | Perrin, 2012 |  |  |  |  |  |
| [57] | Puri, 2012 |  |  |  |  |  |
| [58] | Contreras, 2011 |  |  |  |  |  |
| [59] | Halldén, 2011 |  |  |  |  |  |
| [60] | Harris, 2011 |  |  |  |  |  |
| [61] | Lamichhane, 2011 |  |  |  |  |  |
| [62] | Lindström, 2011 |  |  |  |  |  |
| [63] | Lipp, 2011 |  |  |  |  |  |
| [64] | Mizuno, 2011 |  |  |  |  |  |
| [65] | O’Donnell, 2011 |  |  |  |  |  |
| [66] | Freedman, 2010 |  |  |  |  |  |
| [67] | Gallagher, 2010 |  |  |  |  |  |
| [68] | Lipp, 2010 |  |  |  |  |  |
| [69] | Mamabolo, 2010 |  |  |  |  |  |
| [70] | Graham, 2009 |  |  |  |  |  |
| [71] | Harries, 2009 |  |  |  |  |  |
| **Ref** | **Author, Year** | **1.1 Is the qualitative approach appropriate to answer the research questions?** | **1.2 Are the qualitative data collection methods adequate to address the research questions?** | **1.3 Are the findings adequately derived from the data?** | **1.4 Is the interpretation of results sufficiently substantiated by data?** | **1.5 Is there coherence between qualitative data sources, collection, analysis, and interpretation?** |
| [72] | Lipp, 2008 |  |  |  |  |  |
| [73] | Wolkomir, 2007 |  |  |  |  |  |
| [74] | Statham, 2006 |  |  |  |  |  |
| [75] | Chiappetta-Swanson, 2005 |  |  |  |  |  |
| [76] | Hanna, 2005 |  |  |  |  |  |
| [77] | Mayers, 2005 |  |  |  |  |  |
| [78] | Potgrier, 2004 |  |  |  |  |  |
| [79] | da Costa, 2003 |  |  |  |  |  |
| [80] | Cignacco, 2002 |  |  |  |  |  |
| [81] | Garel, 2002 |  |  |  |  |  |
| [82] | Askey, 2001 |  |  |  |  |  |
| [83] | Gmeimer, 2000 |  |  |  |  |  |

| **Table Legend** | |
| --- | --- |
|  | Yes |
|  | Cannot tell |
|  | No |

**S3 Table 2:** Quantitative Study Design (Non-Randomised)

| **Ref** | **Author, Year** | **3.1 Are the participants representative of the target population?** | **3.2 Are measurements appropriate regarding both the outcome and intervention (or exposure)?** | **3.3 Are there complete outcome data?** | **3.4 Are the confounders accounted for in the design and analysis?** | **3.5 During the study period, is the intervention administered (or exposure occurred) as intended?** |
| --- | --- | --- | --- | --- | --- | --- |
| [84] | Dempsey, 2021 |  |  |  |  |  |
| [85] | Mosley, 2020 |  |  |  |  |  |
| [86] | Zareba, 2020 |  |  |  |  |  |
| [87] | Janiak, 2018 |  |  |  |  |  |
| [88] | Martin, 2014a |  |  |  |  |  |
| [89] | Martin, 2014b |  |  |  |  |  |
| [90] | Mizuno, 2013 |  |  |  |  |  |
| [91] | Turk, 2013 |  |  |  |  |  |
| [92] | Lindström, 2007 |  |  |  |  |  |

| **Table Legend** | |
| --- | --- |
|  | Yes |
|  | Cannot tell |
|  | No |

**S3 Table 3:**  Quantitative Study Design (Descriptive)

| **Ref** | **Author, Year** | **4.1 Is the sampling strategy relevant to address the research question?** | **4.2 Is the sample representative of the target population?** | **4.3 Are the measures appropriate?** | **4.4 Is the risk of nonresponse bias low?** | **4.5 Is the statistical analysis appropriate to answer the research question?** |
| --- | --- | --- | --- | --- | --- | --- |
| [93] | Nkosi, 2020 |  |  |  |  |  |
| [94] | Martin, 2018 |  |  |  |  |  |
| [95] | Puri, 2018 |  |  |  |  |  |
| [96] | Norman, 2013 |  |  |  |  |  |
| [97] | Mokgethi, 2006 |  |  |  |  |  |
| [98] | Donnay, 1993 |  |  |  |  |  |

| **Table Legend** | |
| --- | --- |
|  | Yes |
|  | Cannot tell |
|  | No |

**S3 Table 4:** Mixed Methods Study Design

| **Ref** | **Author. Year** | **5.1 Is there an adequate rationale for using a mixed methods design to address the research question?** | **5.2 Are the different components of the study effectively integrated to answer the research question?** | **5.3 Are the outputs of the integration of qualitative and quantitative components adequately interpreted?** | **5.4 Are divergences and inconsistencies between quantitative and qualitative results adequately addressed?** | **5.5 Do the different components of the study adhere to the quality criteria of each tradition of the methods involved?** |
| --- | --- | --- | --- | --- | --- | --- |
| [99] | Ramón Michel, 2020 |  |  |  |  |  |
| [100] | de Zordo, 2018 |  |  |  |  |  |
| [101] | Teffo, 2017 |  |  |  |  |  |
| [102] | Nicholson, 2010 |  |  |  |  |  |
| [103] | Ordinioha, 2008 |  |  |  |  |  |
| [104] | Garel, 2007 |  |  |  |  |  |
| [105] | Hammarstedt, 2006 |  |  |  |  |  |
| [106] | Fitzpatrick, 1999 |  |  |  |  |  |

| **Table Legend** | |
| --- | --- |
|  | Yes |
|  | Cannot tell |
|  | No |

**References**

1. Chowdhary P, Newton-Levinson A, Rochat R. "No one does this for the money or lifestyle": Abortion providers' perspectives on factors affecting workforce recruitment and retention in the southern United States. Matern Child Health J. 2022;26(6):1350-7.

2. Mainey L, O'Mullan C, Reid-Searl K. Working with or against the system: Nurses' and midwives' process of providing abortion care in the context of gender-based violence in Australia. J Adv Nurs 2022 Epub 2022 March 14 Available from: 101111/jan15226.

3. Newton-Levinson A, Higdon M, Rochat R. Supporting staff in southern family planning clinics: Challenges and opportunities. Matern Child Health J. 2022;26(2):319-27.

4. Reeves JA, Goedken P, Hall KS, Lee SC, Cwiak CA. Anesthesia providers' perspectives on abortion provision: Deductive findings from a qualitative study. Int J Obstet Anesth. 2022;49:103239.

5. Armour S, Gilkison A, Hunter M. Midwives holding the space for women undergoing termination of pregnancy: A qualitative inquiry. Women Birth. 2021;34(6):e616-23.

6. Becker A, Hann LR. "It makes it more real": Examining ambiguous fetal meanings in abortion care. Soc Sci Med. 2021;272:113736.

7. Cannon R, White K, Seifert B, Woodhams E, Brandi K, Yinusa-Nyahkoon L. Exploring the physician's role in contraceptive counseling at the time of abortion in the US. Contraception. 2021;103(5):316-21.

8. Ewnetu DB, Thorsen VC, Solbakk JH, Magelssen M. Navigating abortion law dilemmas: Experiences and attitudes among Ethiopian health care professionals. BMC Med Ethics. 2021;22(1):166.

9. Magelssen M, Ewnetu DB. Professionals' experience with conscientious objection to abortion in Addis Ababa, Ethiopia: An interview study. Dev World Bioeth. 2021;21(2):68-73.

10. McLeod C, Javlekar A, Flink-Bochacki R. Exploring the relationship between abortion provision and providers' personal pregnancy and parenting experiences. WHI. 2021;31(2):171-6.

11. Persson M, Larsson EC, Islam NP, Gemzell-Danielsson K, Klingberg-Allvin M. A qualitative study on health care providers' experiences of providing comprehensive abortion care in Cox's Bazar, Bangladesh. Confl Health. 2021;15(1):6.

12. Rasmussen KN, Janiak E, Cottrill AA, Stulberg DB. Expanding access to medication abortion through pharmacy dispensing of mifepristone: Primary care perspectives from Illinois. Contraception. 2021;104(1):98-103.

13. Simmonds K, Schwartz-Barcott D, Erickson-Owens D. Nurse practitioners' and certified nurse midwives' experiences providing comprehensive early abortion care in New England, USA. Health Care Women Int. 2021;17:1-23.

14. Zwerling B, Rousseau J, Ward KM, Olshansky E, Lo A, Thiel de Bocanegra H, et al. "It's a horrible assignment": A qualitative study of labor and delivery nurses' experience caring for patients undergoing labor induction for fetal anomalies or fetal demise. Contraception. 2021;104(3):301-4.

15. Aborigo RA, Moyer CA, Sekwo E, Kuwolamo I, Kumaga E, Oduro AR, et al. Optimizing task-sharing in abortion care in Ghana: Stakeholder perspectives. Int J Gynecol Obstet. 2020;150(S1):17-24.

16. Ewnetu DB, Thorsen VC, Solbakk JH, Magelssen M. Still a moral dilemma: How Ethiopian professionals providing abortion come to terms with conflicting norms and demands. BMC Med Ethics. 2020;21(1):16.

17. Hasselbacher LA, Hebert LE, Liu Y, Stulberg DB. "My hands are tied": Abortion restrictions and providers' experiences in religious and nonreligious health care systems. Perspect Sex Reprod Health. 2020;52(2):107-15.

18. Mavuso JMJ, Macleod CI. Resisting abortion stigma in situ: South African womxn's and healthcare providers' accounts of the pre-abortion counselling healthcare encounter. Cult Health Sex. 2020;22(11):1299-313.

19. Maxwell KJ, Hoggart L, Bloomer F, Rowlands S, Purcell C. Normalising abortion: What role can health professionals play? BMJ Sex Reprod Health. 2020;47:32-6.

20. Påfs J, Rulisa S, Klingberg-Allvin M, Binder-Finnema P, Musafili A, Essén B. Implementing the liberalized abortion law in Kigali, Rwanda: Ambiguities of rights and responsibilities among health care providers. Midwifery. 2020;80:102568.

21. Power S, Meaney S, O'Donoghue K. Fetal Medicine Specialists' experiences of providing a new service of termination of pregnancy for fatal fetal anomaly: A qualitative study. BJOG. 2020;128(4):676-84.

22. Teffo M, Rispel L. Resilience or detachment? Coping strategies among termination of pregnancy health care providers in two South African provinces. Cult Health Sex. 2020;22(3):336-51.

23. Czarnecki D, Anspach RR, De Vries RG, Dunn MD, Hauschildt K, Harris LH. Conscience reconsidered: The moral work of navigating participation in abortion care on labor and delivery. Soc Sci Med. 2019;232:181-9.

24. Fernández Vázquez SS, Brown J. From stigma to pride: Health professionals and abortion policies in the Metropolitan Area of Buenos Aires. Sex Reprod Health Matters. 2019;27(3):1691898.

25. McLean E, Desalegn DN, Blystad A, Miljeteig I. When the law makes doors slightly open: Ethical dilemmas among abortion service providers in Addis Ababa, Ethiopia. BMC Med Ethics. 2019;20(1):60.

26. Oelhafen S, Monteverde S, Cignacco E. Exploring moral problems and moral competences in midwifery: A qualitative study. Nurs Ethics. 2019;26(5):1373-86.

27. Seewald M, Martin LA, Echeverri L, Njunguru J, Hassinger JA, Harris LH. Stigma and abortion complications: Stories from three continents. Sex Reprod Health Matters. 2019;27(3):1688917.

28. Cárdenas R, Labandera A, Baum SE, Chiribao F, Leus I, Avondet S, et al. "It's something that marks you": Abortion stigma after decriminalization in Uruguay. Reprod Health. 2018;15(1):150.

29. Greenberg S, Nothnagle M. An "invaluable skill": Reflections on abortion training and postresidency practice. Fam Med. 2018;50(9):691-3.

30. Britton LE, Mercier RJ, Buchbinder M, Bryant AG. Abortion providers, professional identity, and restrictive laws: A qualitative study. Health Care Women Int. 2017;38(3):222-37.

31. Dawson AJ, Nicolls R, Bateson D, Doab A, Estoesta J, Brassil A, et al. Medical termination of pregnancy in general practice in Australia: A descriptive-interpretive qualitative study. Reprod Health. 2017;14(1):39.

32. Martin LA, Hassinger JA, Debbink M, Harris LH. Dangertalk: Voices of abortion providers. Soc Sci Med. 2017;184:75-83.

33. Mauri PA, Squillace F. The experience of Italian nurses and midwives in the termination of pregnancy: A qualitative study. Eur J Contracept Reprod Health Care. 2017;22(3):227-32.

34. Purcell C, Cameron S, Lawton J, Glasier A, Harden J. The changing body work of abortion: A qualitative study of the experiences of health professionals. Sociol Health Illn. 2017;39(1):78-94.

35. Aniteye P, O'Brien B, Mayhew SH. Stigmatized by association: Challenges for abortion service providers in Ghana. BMC Health Serv Res. 2016;16(1):486.

36. Debbink MLP, Hassinger JA, Martin LA, Maniere E, Youatt E, Harris LH. Experiences with the Providers Share Workshop method: Abortion worker support and research in tandem. Qual Health Res. 2016;26(13):1823-37.

37. Fay V, Thomas S, Slade P. Maternal-fetal medicine specialists' experiences of conducting feticide as part of termination of pregnancy: A qualitative study. Prenat Diagn. 2016;36(1):92-9.

38. Larsson EC, Fried S, Essén B, Klingberg-Allvin M. Equitable abortion care – A challenge for health care providers. Experiences from abortion care encounters with immigrant women in Stockholm, Sweden. Sex Reprod Healthc. 2016;10:14-8.

39. Purcell C, Cameron S, Lawton J, Glasier A, Harden J. Contraceptive care at the time of medical abortion: Experiences of women and health professionals in a hospital or community sexual and reproductive health context. Contraception. 2016;93(2):170-7.

40. Yang CF, Che HL, Hsieh HW, Wu SM. Concealing emotions: Nurses' experiences with induced abortion care. J Clin Nurs. 2016;25(9-10):1444-54.

41. Black KI, Douglas H, de Costa C. Women's access to abortion after 20 weeks' gestation for fetal chromosomal abnormalities: Views and experiences of doctors in New South Wales and Queensland. Aust N Z J Obstet Gynaecol. 2015;55(2):144-8.

42. Mauri PA, Ceriotti E, Soldi M, Contini NNG. Italian midwives' experiences of late termination of pregnancy. A phenomenological–hermeneutic study. Nurs Health Sci. 2015;17(2):243-9.

43. McLemore MR, Kools S, Levi AJ. Calculus formation: Nurses’ decision‐making in abortion‐related care. Res Nursing Health. 2015;38(3):222-31.

44. McLemore MR, Levi A, Angel James E. Recruitment and retention strategies for expert nurses in abortion care provision. Contraception. 2015;91(6):474-9.

45. Mercier RJ, Buchbinder M, Bryant A, Britton L. The experiences and adaptations of abortion providers practicing under a new TRAP law: A qualitative study. Contraception. 2015;91(6):507-12.

46. Potdar P, Barua A, Dalvie S, Pawar A. "If a woman has even one daughter, I refuse to perform the abortion": Sex determination and safe abortion in India. Reprod Health Matters. 2015;23(45):114-25.

47. Strefling IdSS, Lunardi Filho WD, Kerber NPdC, Soares MC, Ribeiro JP. Nursing perceptions about abortion management and care: A qualitative study. Texto Contexto Enferm. 2015;24(3):784-91.

48. Andersson IM, Gemzell-Danielsson K, Christensson K. Caring for women undergoing second-trimester medical termination of pregnancy. Contraception. 2014;89(5):460-5.

49. Gwangwa TJ, Kgole JC, Matlala F. Experiences of registered midwives performing termination of pregnancy at Polokwane Mankweng Hospital Complex, Limpopo Province, South Africa. Afr J Phys Health Educ Recreat Dance. 2014;1(2):261-74.

50. Parker A, Swanson H, Frunchak V. Needs of labor and delivery nurses caring for women undergoing pregnancy termination. J Obstet Gynecol Neonatal Nurs. 2014;43(4):478-87.

51. Aniteye P, Mayhew SH. Shaping legal abortion provision in Ghana: Using policy theory to understand provider-related obstacles to policy implementation. Health Res Policy Syst. 2013;11:23.

52. Christensen AV, Christiansen AH, Petersson B. Faced with a dilemma: Danish midwives' experiences with and attitudes towards late termination of pregnancy. Scand J Caring Sci. 2013;27(4):913-20.

53. Dressler J, Maughn N, Soon JA, Norman WV. The perspective of rural physicians providing abortion in Canada: qualitative findings of the British Columbia Abortion Providers Survey (BCAPS). PLoS One. 2013;8(6):e67070.

54. Harries J, Lince N, Constant D, Hargey A, Grossman D. The challenges of offering public second trimester abortion services in South Africa: Health care providers' perspectives. J Biosoc Sci. 2012;44(2):197-208.

55. Möller A, Öfverstedt S, Siwe K. Proud, not yet satisfied: The experiences of abortion service providers in the Kathmandu Valley, Nepal. Sex Reprod Healthc. 2012;3(4):135-40.

56. Perrin E, Berthoud M, Pott M, Vera AGT, Bianchi-Demicheli F. Views of healthcare professionals dealing with legal termination of pregnancy up to 12 WA in French-speaking Switzerland. Swiss Med Wkly. 2012;142:w13584.

57. Puri M, Lamichhane P, Harken T, Blum M, Harper CC, Darney PD, et al. "Sometimes they used to whisper in our ears": Health care workers' perceptions of the effects of abortion legalization in Nepal. BMC Public Health. 2012;12.

58. Contreras X, van Dijk MG, Sanchez T, Smith PS. Experiences and opinions of health‐care professionals regarding legal abortion in Mexico City: A qualitative study. Stud Fam Plan. 2011;42(3):183-90.

59. Halldén BM, Lundgren I, Christensson K. Ten Swedish midwives' lived experiences of the care of teenagers' early induced abortions. Health Care Women Int. 2011;32(5):420-40.

60. Harris LH, Debbink M, Martin L, Hassinger J. Dynamics of stigma in abortion work: Findings from a pilot study of the Providers Share Workshop. Soc Sci Med. 2011;73(7):1062-70.

61. Lamichhane P, Harken T, Puri M, Darney PD, Blum M, Harper CC, et al. Sex-selective abortion in Nepal: A qualitative study of health workers' perspectives. WHI. 2011;21(3):S37-S41.

62. Lindström M, Wulff M, Dahlgren L, Lalos A. Experiences of working with induced abortion: Focus group discussions with gynaecologists and midwives/nurses. Scand J Caring Sci. 2011;25(3):542-8.

63. Lipp A. Self-preservation in abortion care: A grounded theory study. J Clin Nurs. 2011;20(5-6):892-900.

64. Mizuno M. Confusion and ethical issues surrounding the role of Japanese midwives in childbirth and abortion: A qualitative study. Nurs Health Sci. 2011;13(4):502-6.

65. O'Donnell J, Weitz TA, Freedman LR. Resistance and vulnerability to stigmatization in abortion work. Soc Sci Med. 2011;73(9):1357-64.

66. Freedman L, Landy U, Darney P, Steinauer J. Obstacles to the integration of abortion into obstetrics and gynecology practice. Perspect Sex Reprod Health. 2010;42(3):146-51.

67. Gallagher K, Porock D, Edgley A. The concept of 'nursing' in the abortion services. J Adv Nurs. 2010;66(4):849-57.

68. Lipp A. Conceding and concealing judgement in termination of pregnancy: A grounded theory study. J Res Nurs. 2010;15(4):365-78.

69. Mamabolo LRC, Tjallinks JE. Experiences of registered nurses at one community health centre near Pretoria providing termination of pregnancy services. Afr J Nurs Midwifery. 2010;12(1):73-86.

70. Graham RH, Mason K, Rankin J, Robson SC. The role of feticide in the context of late termination of pregnancy: A qualitative study of health professionals' and parents' views. Prenat Diagn. 2009;29(9):875-81.

71. Harries J, Stinson K, Orner P. Health care providers' attitudes towards termination of pregnancy: A qualitative study in South Africa. BMC Public Health. 2009;9:296.

72. Lipp A. A woman centred service in termination of pregnancy: A grounded theory study. Contemp Nurse. 2008;31(1):9-19.

73. Wolkomir M, Powers J. Helping women and protecting the self: The challenge of emotional labor in an abortion clinic. Qual Sociol. 2007;30(2):153-69.

74. Statham H, Solomou W, Green J. Late termination of pregnancy: Law, policy and decision making in four English fetal medicine units. BJOG. 2006;113(12):1402-11.

75. Chiappetta-Swanson C. Dignity and dirty work: Nurses' experiences in managing genetic termination for fetal anomaly. Qual Sociol. 2005;28(1):93-116.

76. Hanna DR. The lived experience of moral distress: Nurses who assisted with elective abortions. Res Theory Nurs Pract. 2005;19(1):95-124.

77. Mayers PM, Parkes B, Green B, Turner J. Experiences of registered midwives assisting with termination of pregnancies at a tertiary level hospital. Health SA Gesondheid. 2005;10(1):15-25.

78. Potgrier C, Andrews G. South African nurses' accounts for choosing to be termination of pregnancy providers. Health SA Gesondheid. 2004;9(2):20-30.

79. da Costa PC, Donald F. The experience of person-role conflict in doctors expected to terminate pregnancies in the South African public sector. S Afr J Psychol. 2003;33(1):10-8.

80. Cignacco E. Between professional duty and ethical confusion: Midwives and selective termination of pregnancy. Nurs Ethics. 2002;9(2):179-91; discussion 91-3.

81. Garel M, Gosme-Seguret S, Kaminski M, Cuttini M. Ethical decision-making in prenatal diagnosis and termination of pregnancy: A qualitative survey among physicians and midwives. Prenat Diagn. 2002;22(9):811-7.

82. Askey K, Moss L. Termination for fetal defects: The effect on midwifery staff. Br J Midwifery. 2001;9(1):17-24.

83. Gmeiner AC, Van Wyk S, Poggenpoel M, Myburgh CP. Support for nurses directly involved with women who chose to terminate a pregnancy. Curationis. 2000;23(1):70-8.

84. Dempsey B, Favier M, Mullally A, Higgins MF. Exploring providers' experience of stigma following the introduction of more liberal abortion care in the Republic of Ireland. Contraception. 2021;104(4):414-9.

85. Mosley EA, Martin L, Seewald M, Hassinger J, Blanchard K, Baum SE, et al. Addressing abortion provider stigma: A pilot implementation of the Providers Share Workshop in Sub-Saharan Africa and Latin America. Int Perspect Sex Reprod Health. 2020;46:35-50.

86. Zaręba K, Banasiewicz J, Rozenek H, Ciebiera M, Jakiel G. Emotional complications in midwives participating in pregnancy termination procedure: Polish experience. Int J Environ Res Public Health. 2020;17(8).

87. Janiak E, Freeman S, Maurer R, Berkman LF, Goldberg AB, Bartz D. Relationship of job role and clinic type to perceived stigma and occupational stress among abortion workers. Contraception. 2018;98(6):517-21.

88. Martin LA, Debbink M, Hassinger J, Youatt E, Eagen-Torkko M, Harris LH. Measuring stigma among abortion providers: Assessing the Abortion Provider Stigma Survey. Women Health. 2014;54(7):641-61.

89. Martin LA, Debbink M, Hassinger J, Youatt E, Harris LH. Abortion providers, stigma and professional quality of life. Contraception. 2014;90(6):581-7.

90. Mizuno M, Kinefuchi E, Kimura R, Tsuda A. Professional quality of life of Japanese nurses/midwives providing abortion/childbirth care. Nurs Ethics. 2013;20(5):539-50.

91. Turk JK, Steinauer JE, Landy U, Kerns JL. Barriers to D&E practice among family planning subspecialists. Contraception. 2013;88(4):561-7.

92. Lindström M, Jacobsson L, Wulff M, Lalos A. Midwives' experiences of encountering women seeking an abortion. J Psychosom Obstet Gynaecol. 2007;28(4):231-7.

93. Nkosi LJ, Mulaudzi FM, Peu MD. Challenges related to the structure of the choice on termination of pregnancy services in public health facilities in the Tshwane District of Gauteng. Afr J Reprod Health. 2020;24(1):106-14.

94. Martin LA, Hassinger JA, Seewald M, Harris LH. Evaluation of abortion stigma in the workforce: Development of the Rrevised Abortion Providers Stigma Scale. WHI. 2018;28(1):59-67.

95. Puri MC, Raifman S, Khanal B, Maharjan DC, Foster DG. Providers' perspectives on denial of abortion care in Nepal: A cross sectional study. Reprod Health. 2018;15(1):170.

96. Norman WV, Soon JA, Maughn N, Dressler J. Barriers to rural induced abortion services in Canada: findings of the British Columbia Abortion Providers Survey (BCAPS). PLoS One. 2013;8(6):e67023.

97. Mokgethi NE, Ehlers VJ, van der Merwe MM. Professional nurses' attitudes towards providing termination of pregnancy services in a tertiary hospital in the north west province of South Africa. Curationis. 2006;29(1):32-9.

98. Donnay F, Bregentzer A, Leemans P, Verougstraete A, Vekemans M. Safe abortions in an illegal context: Perceptions from service providers in Belgium. Stud Fam Plann. 1993;24(3):150-62.

99. Ramón Michel A, Kung S, López-Salm A, Ariza Navarrete S. Regulating conscientious objection to legal abortion in Argentina - Taking into consideration its uses and consequences. Health Hum Rights. 2020;22(2):271-83.

100. De Zordo S. From women's 'irresponsibility' to foetal 'patienthood': Obstetricians-gynaecologists' perspectives on abortion and its stigmatisation in Italy and Cataluña. Glob Public Health. 2018;13(6):711-23.

101. Teffo ME, Rispel LC. 'I am all alone': Factors influencing the provision of termination of pregnancy services in two South African provinces. Glob Health Action. 2017;10(1):1347369.

102. Nicholson J, Slade P, Fletcher J. Termination of pregnancy services: Experiences of gynaecological nurses. J Adv Nurs. 2010;66(10):2245-56.

103. Ordinioha B, Brisibe S. Clandestine abortion in Port Harcourt: providers' motivations and experiences. Niger J Med. 2008;17(3):291-5.

104. Garel M, Etienne E, Blondel B, Dommergues M. French midwives' practice of termination of pregnancy for fetal abnormality. At what psychological and ethical cost? Prenat Diagn. 2007;27(7):622-8.

105. Hammarstedt M, Lalos A, Wulff M. A population-based study of Swedish gynecologists' experiences of working in abortion care. Acta Obstet Gynecol Scand. 2006;85(2):229-35.

106. Fitzpatrick KM, Wilson M. Exposure to violence and posttraumatic stress symptomatology among abortion clinic workers. J Trauma Stress. 1999;12(2):227-42.
