# Supplementary material for: Providers’ experiences with abortion care: A scoping review
Source: PLoS One. 2024 Jul 1;19(7):e0303601. doi: 10.1371/journal.pone.0303601 (PMC11216598; doi:10.1371/journal.pone.0303601)
Supplement: S4 Table — (DOCX) [file pone.0303601.s004.docx]

**S4 Table**

**Overview of the laws governing abortion care in each of the countries studied in the scoping review on providers’ experiences with abortion care**

**S4 Table:** Overview of legislation, adapted from a United Nations report on legal grounds for abortion in each country [1].

| **Continent**  **Country** | **Risk to Life** | **Risk to Physical Health** | **Risk to Mental Health** | **Cases of Incest/Rape** | **Fetal Abnormality** | **Economic or Social Reasons** | **On Request** | **Studies** |
| --- | --- | --- | --- | --- | --- | --- | --- | --- |
| **Africa (24 Studies)** | |  |  |  |  |  |  |  |
| **Ethiopia** | **28 weeks** | **28 weeks** | **28 weeks** | **28 weeks** | **28 weeks** | **Illegal** | **Illegal** | 4 |
| **Ghana** | **28 weeks** | **28 weeks** | **28 weeks** | **28 weeks** | **28 weeks** | **Illegal** | **Illegal** | 3 |
| **Nigeria** | **No Limit** | **No Limit** | **No Limit** | **Illegal** | **N/A** | **Illegal** | **Illegal** | 1 |
| **Rwanda** | **No Limit** | **No Limit** | **No Limit** | **22 weeks** | **No Limit** | **Illegal** | **Illegal** | 1 |
| **South Africa** | **No Limit** | **20 weeks** | **20 weeks** | **20 weeks** | **No Limit** | **20 weeks** | **12 weeks** | 13 |
| **Three unnamed countries** | **N/A** | **N/A** | **N/A** | **N/A** | **N/A** | **N/A** | **N/A** | [2, 3] |
|  |  |  |  |  |  |  |  |  |
| **Asia (9 Studies)** | |  |  |  |  |  |  |  |
| **Bangladesh** | **No Limit** | **Illegal** | **Illegal** | **Illegal** | **Illegal** | **Illegal** | **Illegal** | 1 |
| **India** | **No Limit** | **24 weeks** | **24 weeks** | **24 weeks** | **24 weeks** | **24 weeks** | **N/A** | 1 |
| **Japan** | **22 weeks** | **22 weeks** | **22 weeks** | **22 weeks** | **Illegal** | **22 weeks** | **Illegal** | 2 |
| **Nepal** | **28 weeks** | **28 weeks** | **28 weeks** | **28 weeks** | **28 weeks** | **12 weeks** | **12 weeks** | 4 |
| **Taiwan** | **No Limit** | **No Limit** | **No Limit** | **24 weeks** | **No Limit** | **24 weeks** | **Illegal** | 1 |
|  |  |  |  |  |  |  |  |  |
| **Oceania (4 Studies)** | |  |  |  |  |  |  |  |
| **Australia** | **No Limit** | **No Limit** | **No Limit** | **No Limit** | **No Limit** | **No Limit** | **No Limit** | 3 |
| **New Zealand** | **No Limit** | **No Limit** | **No Limit** | **No Limit** | **No Limit** | **No Limit** | **20 weeks** | 1 |
|  |  |  |  |  |  |  |  |  |
| **Europe (31 Studies)** | |  |  |  |  |  |  |  |
| **Belgium** | **No Limit** | **No Limit** | **No Limit** | **12 weeks** | **No Limit** | **12 weeks** | **12 weeks** | 1 |
| **Denmark** | **No Limit** | **No Limit** | **No Limit** | **No Limit** | **No Limit** | **12 weeks** | **12 weeks** | 1 |
| **France** | **No Limit** | **No Limit** | **No Limit** | **14 weeks** | **No Limit** | **14 weeks** | **14 weeks** | 2 |

| **Continent**  **Country** | **Risk to Life** | **Risk to Physical Health** | **Risk to Mental Health** | **Cases of Incest/Rape** | **Fetal Abnormality** | **Economic or Social Reasons** | **On Request** | **Studies** |
| --- | --- | --- | --- | --- | --- | --- | --- | --- |
| **Europe cont. (31 Studies)** | |  |  |  |  |  |  |  |
| **Ireland, Republic of** | **No Limit** | **No Limit** | **No Limit** | **12 weeks** | **No Limit** | **12 weeks** | **12 weeks** | 1 |
| **Italy** | **No Limit** | **No Limit** | **No Limit** | **13 weeks** | **13 weeks** | **13 weeks** | **13 weeks** | 3 |
| **Poland** | **No Limit** | **No Limit** | **No Limit** | **13 weeks** | **Illegal** | **Illegal** | **Illegal** | 1 |
| **Spain** | **22 weeks** | **22 weeks** | **22 weeks** | **14 weeks** | **22 weeks** | **14 weeks** | **14 weeks** | 1 |
| **Sweden** | **No Limit** | **No Limit** | **No Limit** | **18 weeks** | **18 weeks** | **18 weeks** | **18 weeks** | 6 |
| **Switzerland** | **No Limit** | **No Limit** | **No Limit** | **12 weeks** | **12 weeks** | **12 weeks** | **12 weeks** | 3 |
| **UK (excl. Northern Ireland)** | **No Limit** | **No Limit** | **No Limit** | **N/A** | **No Limit** | **24 weeks** | **Illegal** | 12 |
|  |  |  |  |  |  |  |  |  |
| **North America (35 Studies)** | |  |  |  |  |  |  |  |
| **Canada** | **No Limit** | **No Limit** | **No Limit** | **No Limit** | **No Limit** | **No Limit** | **No Limit** | 4 |
| **Mexico** | **No Limit** | **20 weeks** | **20 weeks** | **20 weeks** | **20 weeks** | **12 weeks** | **12 weeks** | 1 |
| **USA** | **No Limit** | **No Limit** | **Varies by State** | **Varies by State** | **Varies by State** | **Varies by State** | **Varies by State** | 29 |
|  |  |  |  |  |  |  |  |  |
| **South America (6 Studies)** | |  |  |  |  |  |  |  |
| **Argentina** | **No Limit** | **No Limit** | **No Limit** | **No Limit** | **14 weeks** | **14 weeks** | **14 weeks** | 2 |
| **Brazil** | **22 weeks** | **Illegal** | **Illegal** | **22 weeks** | **Illegal** | **Illegal** | **Illegal** | 1 |
| **Uruguay** | **No Limit** | **No Limit** | **No Limit** | **14 weeks** | **No Limit** | **12 weeks** | **12 weeks** | 1 |
| **Seven unnamed countries** | **N/A** | **N/A** | **N/A** | **N/A** | **N/A** | **N/A** | **N/A** | [2, 3] |

| **Table Legend** | |
| --- | --- |
|  | Abortion legally permitted in accordance with gestational age limit |
|  | Legal for cases of rape or incest, but not both |
|  | Legal status of abortion varies by states, see resource for more information [4] |
|  | Abortions is legally prohibited for these reasons |
|  | No data available to the United Nations Team |

**References**

1. United Nations. Countries by legal grounds for abortion. 2019 [Available from: <https://www.un.org/en/development/desa/population/theme/policy/GAPP_country_data.xlsx> (Accessed on 24th of August 2022).

2. Mosley EA, Martin L, Seewald M, Hassinger J, Blanchard K, Baum SE, et al. Addressing abortion provider stigma: A pilot implementation of the Providers Share Workshop in Sub-Saharan Africa and Latin America. Int Perspect Sex Reprod Health. 2020;46:35-50.

3. Seewald M, Martin LA, Echeverri L, Njunguru J, Hassinger JA, Harris LH. Stigma and abortion complications: Stories from three continents. Sex Reprod Health Matters. 2019;27(3):1688917.

4. Guttmacher Institute. Interactive Map: US abortion policies and access after Roe [Available from: <https://states.guttmacher.org/policies/california/abortion-policies> (Accessed on 24th of August, 2022).
