# Supplementary material for: Providers’ experiences with abortion care: A scoping review
Source: PLoS One. 2024 Jul 1;19(7):e0303601. doi: 10.1371/journal.pone.0303601 (PMC11216598; doi:10.1371/journal.pone.0303601)
Supplement: S5 Table — (DOCX) [file pone.0303601.s005.docx]

**S5 Table**

**Overview of descriptive and analytical themes devised as part of the scoping review on providers’ experiences with abortion care with supporting references**

**Table 5A:** Overview of Scoping Review Themes with Supporting References

| **Theme** | **Sub-Theme** | **Aspects of the Sub-Theme** | **Supporting References** |
| --- | --- | --- | --- |
| **Providers’ experiences with abortion stigma** | *Within society* | Laws governing abortion care | [1-32] |
|  |  | Anti-abortion sentiments in the media | [11, 12, 20, 23, 33-35] |
|  | *Within the community* | Anti-abortion sentiments in the community | [1, 2, 5-8, 11-23, 25, 26, 29-31, 33-67] |
|  |  | Anti-abortion sentiments from family and friends | [5, 6, 14, 17, 21, 22, 36, 38, 41, 42, 50, 52-54] |
|  |  | Hiding abortion work | [2, 5, 6, 8, 11, 14, 15, 17, 19, 21, 22, 30, 33, 36, 39, 41-44, 50, 52-55, 58, 62] |
|  | *Within the workplace* | Negative experiences with colleagues | [2, 3, 5, 6, 8, 14-17, 20-23, 26, 33, 34, 36, 38, 41-43, 45, 47, 48, 50-55, 58, 60, 61, 64, 65, 67-84] |
|  |  | Positive experiences with colleagues | [5, 6, 15, 17, 21-24, 29, 30, 35, 38, 40-43, 48, 50, 52, 54, 57, 58, 61, 62, 68, 69, 72-75, 77, 82, 83, 85-90] |
|  |  | Anti-abortion sentiments from patients | [5, 6, 13, 18, 35, 50, 52-54, 66, 74] |
|  |  | Under-resourced services | [2-4, 8, 14, 15, 19, 20, 22, 23, 25-27, 30, 34, 38, 39, 41, 43, 45, 47, 48, 50, 54, 55, 58, 60, 65, 68-70, 74, 75, 77, 79, 83-89, 91-94] |
| **Providers’ reflections on their abortion work** | *Providers’ views on abortion* | | [1, 2, 4, 6-8, 13-15, 17, 18, 21-28, 30-32, 34, 35, 40, 41, 44, 47-50, 52-56, 58, 61-64, 66, 68, 71-75, 77-87, 89, 90, 92, 93, 95-104] |
|  | *Challenging aspects of abortion care* | The fetus | [6, 9, 13, 21-24, 26, 28, 35, 40, 44, 47, 50, 56, 58, 61, 62, 64, 70, 73, 74, 77, 79-87, 89, 90, 92, 94, 98, 99, 101, 103, 105] |
|  |  | Medical vs surgical care | [2, 4, 34, 35, 40, 41, 47, 55, 61, 64, 67, 70, 76, 82-84, 96, 101] |
|  |  | Value judgments about patients | [2, 6, 9, 12-14, 21, 22, 24-26, 28, 31, 34, 40, 44, 47, 50, 54, 56, 58, 61, 63, 70, 73, 74, 78, 79, 82, 83, 85, 87, 89, 95, 96, 98, 100, 102-104] |
|  | *Providers’ emotional responses* | Difficult emotions in abortion work | [2, 4-6, 9, 13, 14, 21-23, 30, 34, 36, 37, 39, 42, 44, 46-48, 50, 54-57, 59, 61-64, 66-68, 72-75, 77, 79-87, 89, 90, 92-94, 96, 97, 103, 105] |
|  |  | Positive emotions in abortion work | [1, 4, 5, 17, 21, 22, 27, 31, 33, 36, 37, 47, 52, 54, 55, 58, 60, 61, 64, 66, 67, 72-75, 77, 84-87, 101, 103] |

**References**

1. Britton LE, Mercier RJ, Buchbinder M, Bryant AG. Abortion providers, professional identity, and restrictive laws: A qualitative study. Health Care Women Int. 2017;38(3):222-37.

2. Cárdenas R, Labandera A, Baum SE, Chiribao F, Leus I, Avondet S, et al. "It's something that marks you": Abortion stigma after decriminalization in Uruguay. Reprod Health. 2018;15(1):150.

3. Black KI, Douglas H, de Costa C. Women's access to abortion after 20 weeks' gestation for fetal chromosomal abnormalities: Views and experiences of doctors in New South Wales and Queensland. Aust N Z J Obstet Gynaecol. 2015;55(2):144-8.

4. Greenberg S, Nothnagle M. An "invaluable skill": Reflections on abortion training and postresidency practice. Fam Med. 2018;50(9):691-3.

5. Harris LH, Debbink M, Martin L, Hassinger J. Dynamics of stigma in abortion work: Findings from a pilot study of the Providers Share Workshop. Soc Sci Med. 2011;73(7):1062-70.

6. Martin LA, Hassinger JA, Debbink M, Harris LH. Dangertalk: Voices of abortion providers. Soc Sci Med. 2017;184:75-83.

7. Mercier RJ, Buchbinder M, Bryant A, Britton L. The experiences and adaptations of abortion providers practicing under a new TRAP law: A qualitative study. Contraception. 2015;91(6):507-12.

8. Påfs J, Rulisa S, Klingberg-Allvin M, Binder-Finnema P, Musafili A, Essén B. Implementing the liberalized abortion law in Kigali, Rwanda: Ambiguities of rights and responsibilities among health care providers. Midwifery. 2020;80:102568.

9. Donnay F, Bregentzer A, Leemans P, Verougstraete A, Vekemans M. Safe abortions in an illegal context: Perceptions from service providers in Belgium. Stud Fam Plann. 1993;24(3):150-62.

10. Ordinioha B, Brisibe S. Clandestine abortion in Port Harcourt: providers' motivations and experiences. Niger J Med. 2008;17(3):291-5.

11. Seewald M, Martin LA, Echeverri L, Njunguru J, Hassinger JA, Harris LH. Stigma and abortion complications: Stories from three continents. Sex Reprod Health Matters. 2019;27(3):1688917.

12. Potdar P, Barua A, Dalvie S, Pawar A. "If a woman has even one daughter, I refuse to perform the abortion": Sex determination and safe abortion in India. Reprod Health Matters. 2015;23(45):114-25.

13. da Costa PC, Donald F. The experience of person-role conflict in doctors expected to terminate pregnancies in the South African public sector. S Afr J Psychol. 2003;33(1):10-8.

14. McLean E, Desalegn DN, Blystad A, Miljeteig I. When the law makes doors slightly open: Ethical dilemmas among abortion service providers in Addis Ababa, Ethiopia. BMC Med Ethics. 2019;20(1):60.

15. Chowdhary P, Newton-Levinson A, Rochat R. "No one does this for the money or lifestyle": Abortion providers' perspectives on factors affecting workforce recruitment and retention in the southern United States. Matern Child Health J. 2022;26(6):1350-7.

16. Rasmussen KN, Janiak E, Cottrill AA, Stulberg DB. Expanding access to medication abortion through pharmacy dispensing of mifepristone: Primary care perspectives from Illinois. Contraception. 2021;104(1):98-103.

17. Simmonds K, Schwartz-Barcott D, Erickson-Owens D. Nurse practitioners' and certified nurse midwives' experiences providing comprehensive early abortion care in New England, USA. Health Care Women Int. 2021;17:1-23.

18. Strefling IdSS, Lunardi Filho WD, Kerber NPdC, Soares MC, Ribeiro JP. Nursing perceptions about abortion management and care: A qualitative study. Texto Contexto Enferm. 2015;24(3):784-91.

19. Aniteye P, O'Brien B, Mayhew SH. Stigmatized by association: Challenges for abortion service providers in Ghana. BMC Health Serv Res. 2016;16(1):486.

20. Contreras X, van Dijk MG, Sanchez T, Smith PS. Experiences and opinions of health‐care professionals regarding legal abortion in Mexico City: A qualitative study. Stud Fam Plan. 2011;42(3):183-90.

21. Fay V, Thomas S, Slade P. Maternal-fetal medicine specialists' experiences of conducting feticide as part of termination of pregnancy: A qualitative study. Prenat Diagn. 2016;36(1):92-9.

22. McLemore MR, Kools S, Levi AJ. Calculus formation: Nurses’ decision‐making in abortion‐related care. Res Nursing Health. 2015;38(3):222-31.

23. Power S, Meaney S, O'Donoghue K. Fetal Medicine Specialists' experiences of providing a new service of termination of pregnancy for fatal fetal anomaly: A qualitative study. BJOG. 2020;128(4):676-84.

24. Statham H, Solomou W, Green J. Late termination of pregnancy: Law, policy and decision making in four English fetal medicine units. BJOG. 2006;113(12):1402-11.

25. Puri M, Lamichhane P, Harken T, Blum M, Harper CC, Darney PD, et al. "Sometimes they used to whisper in our ears": Health care workers' perceptions of the effects of abortion legalization in Nepal. BMC Public Health. 2012;12.

26. Harries J, Stinson K, Orner P. Health care providers' attitudes towards termination of pregnancy: A qualitative study in South Africa. BMC Public Health. 2009;9:296.

27. Persson M, Larsson EC, Islam NP, Gemzell-Danielsson K, Klingberg-Allvin M. A qualitative study on health care providers' experiences of providing comprehensive abortion care in Cox's Bazar, Bangladesh. Confl Health. 2021;15(1):6.

28. Ewnetu DB, Thorsen VC, Solbakk JH, Magelssen M. Navigating abortion law dilemmas: Experiences and attitudes among Ethiopian health care professionals. BMC Med Ethics. 2021;22(1):166.

29. Fernández Vázquez SS, Brown J. From stigma to pride: Health professionals and abortion policies in the Metropolitan Area of Buenos Aires. Sex Reprod Health Matters. 2019;27(3):1691898.

30. Gmeiner AC, Van Wyk S, Poggenpoel M, Myburgh CP. Support for nurses directly involved with women who chose to terminate a pregnancy. Curationis. 2000;23(1):70-8.

31. Möller A, Öfverstedt S, Siwe K. Proud, not yet satisfied: The experiences of abortion service providers in the Kathmandu Valley, Nepal. Sex Reprod Healthc. 2012;3(4):135-40.

32. Potgrier C, Andrews G. South African nurses' accounts for choosing to be termination of pregnancy providers. Health SA Gesondheid. 2004;9(2):20-30.

33. Martin LA, Debbink M, Hassinger J, Youatt E, Eagen-Torkko M, Harris LH. Measuring stigma among abortion providers: Assessing the Abortion Provider Stigma Survey. Women Health. 2014;54(7):641-61.

34. De Zordo S. From women's 'irresponsibility' to foetal 'patienthood': Obstetricians-gynaecologists' perspectives on abortion and its stigmatisation in Italy and Cataluña. Glob Public Health. 2018;13(6):711-23.

35. Gallagher K, Porock D, Edgley A. The concept of 'nursing' in the abortion services. J Adv Nurs. 2010;66(4):849-57.

36. Martin LA, Hassinger JA, Seewald M, Harris LH. Evaluation of abortion stigma in the workforce: Development of the Rrevised Abortion Providers Stigma Scale. WHI. 2018;28(1):59-67.

37. Martin LA, Debbink M, Hassinger J, Youatt E, Harris LH. Abortion providers, stigma and professional quality of life. Contraception. 2014;90(6):581-7.

38. Aborigo RA, Moyer CA, Sekwo E, Kuwolamo I, Kumaga E, Oduro AR, et al. Optimizing task-sharing in abortion care in Ghana: Stakeholder perspectives. Int J Gynecol Obstet. 2020;150(S1):17-24.

39. Aniteye P, Mayhew SH. Shaping legal abortion provision in Ghana: Using policy theory to understand provider-related obstacles to policy implementation. Health Res Policy Syst. 2013;11:23.

40. Czarnecki D, Anspach RR, De Vries RG, Dunn MD, Hauschildt K, Harris LH. Conscience reconsidered: The moral work of navigating participation in abortion care on labor and delivery. Soc Sci Med. 2019;232:181-9.

41. Dawson AJ, Nicolls R, Bateson D, Doab A, Estoesta J, Brassil A, et al. Medical termination of pregnancy in general practice in Australia: A descriptive-interpretive qualitative study. Reprod Health. 2017;14(1):39.

42. Debbink MLP, Hassinger JA, Martin LA, Maniere E, Youatt E, Harris LH. Experiences with the Providers Share Workshop method: Abortion worker support and research in tandem. Qual Health Res. 2016;26(13):1823-37.

43. Dressler J, Maughn N, Soon JA, Norman WV. The perspective of rural physicians providing abortion in Canada: qualitative findings of the British Columbia Abortion Providers Survey (BCAPS). PLoS One. 2013;8(6):e67070.

44. Ewnetu DB, Thorsen VC, Solbakk JH, Magelssen M. Still a moral dilemma: How Ethiopian professionals providing abortion come to terms with conflicting norms and demands. BMC Med Ethics. 2020;21(1):16.

45. Hasselbacher LA, Hebert LE, Liu Y, Stulberg DB. "My hands are tied": Abortion restrictions and providers' experiences in religious and nonreligious health care systems. Perspect Sex Reprod Health. 2020;52(2):107-15.

46. Janiak E, Freeman S, Maurer R, Berkman LF, Goldberg AB, Bartz D. Relationship of job role and clinic type to perceived stigma and occupational stress among abortion workers. Contraception. 2018;98(6):517-21.

47. Lindström M, Jacobsson L, Wulff M, Lalos A. Midwives' experiences of encountering women seeking an abortion. J Psychosom Obstet Gynaecol. 2007;28(4):231-7.

48. Mamabolo LRC, Tjallinks JE. Experiences of registered nurses at one community health centre near Pretoria providing termination of pregnancy services. Afr J Nurs Midwifery. 2010;12(1):73-86.

49. Mavuso JMJ, Macleod CI. Resisting abortion stigma in situ: South African womxn's and healthcare providers' accounts of the pre-abortion counselling healthcare encounter. Cult Health Sex. 2020;22(11):1299-313.

50. Mayers PM, Parkes B, Green B, Turner J. Experiences of registered midwives assisting with termination of pregnancies at a tertiary level hospital. Health SA Gesondheid. 2005;10(1):15-25.

51. Norman WV, Soon JA, Maughn N, Dressler J. Barriers to rural induced abortion services in Canada: findings of the British Columbia Abortion Providers Survey (BCAPS). PLoS One. 2013;8(6):e67023.

52. O'Donnell J, Weitz TA, Freedman LR. Resistance and vulnerability to stigmatization in abortion work. Soc Sci Med. 2011;73(9):1357-64.

53. Maxwell KJ, Hoggart L, Bloomer F, Rowlands S, Purcell C. Normalising abortion: What role can health professionals play? BMJ Sex Reprod Health. 2020;47:32-6.

54. Teffo M, Rispel L. Resilience or detachment? Coping strategies among termination of pregnancy health care providers in two South African provinces. Cult Health Sex. 2020;22(3):336-51.

55. Teffo ME, Rispel LC. 'I am all alone': Factors influencing the provision of termination of pregnancy services in two South African provinces. Glob Health Action. 2017;10(1):1347369.

56. Yang CF, Che HL, Hsieh HW, Wu SM. Concealing emotions: Nurses' experiences with induced abortion care. J Clin Nurs. 2016;25(9-10):1444-54.

57. Mosley EA, Martin L, Seewald M, Hassinger J, Blanchard K, Baum SE, et al. Addressing abortion provider stigma: A pilot implementation of the Providers Share Workshop in Sub-Saharan Africa and Latin America. Int Perspect Sex Reprod Health. 2020;46:35-50.

58. Chiappetta-Swanson C. Dignity and dirty work: Nurses' experiences in managing genetic termination for fetal anomaly. Qual Sociol. 2005;28(1):93-116.

59. Fitzpatrick KM, Wilson M. Exposure to violence and posttraumatic stress symptomatology among abortion clinic workers. J Trauma Stress. 1999;12(2):227-42.

60. Gwangwa TJ, Kgole JC, Matlala F. Experiences of registered midwives performing termination of pregnancy at Polokwane Mankweng Hospital Complex, Limpopo Province, South Africa. Afr J Phys Health Educ Recreat Dance. 2014;1(2):261-74.

61. Hanna DR. The lived experience of moral distress: Nurses who assisted with elective abortions. Res Theory Nurs Pract. 2005;19(1):95-124.

62. Becker A, Hann LR. "It makes it more real": Examining ambiguous fetal meanings in abortion care. Soc Sci Med. 2021;272:113736.

63. Cannon R, White K, Seifert B, Woodhams E, Brandi K, Yinusa-Nyahkoon L. Exploring the physician's role in contraceptive counseling at the time of abortion in the US. Contraception. 2021;103(5):316-21.

64. McLeod C, Javlekar A, Flink-Bochacki R. Exploring the relationship between abortion provision and providers' personal pregnancy and parenting experiences. WHI. 2021;31(2):171-6.

65. Newton-Levinson A, Higdon M, Rochat R. Supporting staff in southern family planning clinics: Challenges and opportunities. Matern Child Health J. 2022;26(2):319-27.

66. Wolkomir M, Powers J. Helping women and protecting the self: The challenge of emotional labor in an abortion clinic. Qual Sociol. 2007;30(2):153-69.

67. Dempsey B, Favier M, Mullally A, Higgins MF. Exploring providers' experience of stigma following the introduction of more liberal abortion care in the Republic of Ireland. Contraception. 2021;104(4):414-9.

68. McLemore MR, Levi A, Angel James E. Recruitment and retention strategies for expert nurses in abortion care provision. Contraception. 2015;91(6):474-9.

69. Freedman L, Landy U, Darney P, Steinauer J. Obstacles to the integration of abortion into obstetrics and gynecology practice. Perspect Sex Reprod Health. 2010;42(3):146-51.

70. Harries J, Lince N, Constant D, Hargey A, Grossman D. The challenges of offering public second trimester abortion services in South Africa: Health care providers' perspectives. J Biosoc Sci. 2012;44(2):197-208.

71. Ramón Michel A, Kung S, López-Salm A, Ariza Navarrete S. Regulating conscientious objection to legal abortion in Argentina - Taking into consideration its uses and consequences. Health Hum Rights. 2020;22(2):271-83.

72. Mainey L, O'Mullan C, Reid-Searl K. Working with or against the system: Nurses' and midwives' process of providing abortion care in the context of gender-based violence in Australia. J Adv Nurs 2022 Epub 2022 March 14 Available from: 101111/jan15226.

73. Hammarstedt M, Lalos A, Wulff M. A population-based study of Swedish gynecologists' experiences of working in abortion care. Acta Obstet Gynecol Scand. 2006;85(2):229-35.

74. Nicholson J, Slade P, Fletcher J. Termination of pregnancy services: Experiences of gynaecological nurses. J Adv Nurs. 2010;66(10):2245-56.

75. Parker A, Swanson H, Frunchak V. Needs of labor and delivery nurses caring for women undergoing pregnancy termination. J Obstet Gynecol Neonatal Nurs. 2014;43(4):478-87.

76. Turk JK, Steinauer JE, Landy U, Kerns JL. Barriers to D&E practice among family planning subspecialists. Contraception. 2013;88(4):561-7.

77. Armour S, Gilkison A, Hunter M. Midwives holding the space for women undergoing termination of pregnancy: A qualitative inquiry. Women Birth. 2021;34(6):e616-23.

78. Magelssen M, Ewnetu DB. Professionals' experience with conscientious objection to abortion in Addis Ababa, Ethiopia: An interview study. Dev World Bioeth. 2021;21(2):68-73.

79. Zwerling B, Rousseau J, Ward KM, Olshansky E, Lo A, Thiel de Bocanegra H, et al. "It's a horrible assignment": A qualitative study of labor and delivery nurses' experience caring for patients undergoing labor induction for fetal anomalies or fetal demise. Contraception. 2021;104(3):301-4.

80. Christensen AV, Christiansen AH, Petersson B. Faced with a dilemma: Danish midwives' experiences with and attitudes towards late termination of pregnancy. Scand J Caring Sci. 2013;27(4):913-20.

81. Cignacco E. Between professional duty and ethical confusion: Midwives and selective termination of pregnancy. Nurs Ethics. 2002;9(2):179-91; discussion 91-3.

82. Lindström M, Wulff M, Dahlgren L, Lalos A. Experiences of working with induced abortion: Focus group discussions with gynaecologists and midwives/nurses. Scand J Caring Sci. 2011;25(3):542-8.

83. Mauri PA, Squillace F. The experience of Italian nurses and midwives in the termination of pregnancy: A qualitative study. Eur J Contracept Reprod Health Care. 2017;22(3):227-32.

84. Purcell C, Cameron S, Lawton J, Glasier A, Harden J. The changing body work of abortion: A qualitative study of the experiences of health professionals. Sociol Health Illn. 2017;39(1):78-94.

85. Andersson IM, Gemzell-Danielsson K, Christensson K. Caring for women undergoing second-trimester medical termination of pregnancy. Contraception. 2014;89(5):460-5.

86. Garel M, Etienne E, Blondel B, Dommergues M. French midwives' practice of termination of pregnancy for fetal abnormality. At what psychological and ethical cost? Prenat Diagn. 2007;27(7):622-8.

87. Mauri PA, Ceriotti E, Soldi M, Contini NNG. Italian midwives' experiences of late termination of pregnancy. A phenomenological–hermeneutic study. Nurs Health Sci. 2015;17(2):243-9.

88. Mokgethi NE, Ehlers VJ, van der Merwe MM. Professional nurses' attitudes towards providing termination of pregnancy services in a tertiary hospital in the north west province of South Africa. Curationis. 2006;29(1):32-9.

89. Garel M, Gosme-Seguret S, Kaminski M, Cuttini M. Ethical decision-making in prenatal diagnosis and termination of pregnancy: A qualitative survey among physicians and midwives. Prenat Diagn. 2002;22(9):811-7.

90. Askey K, Moss L. Termination for fetal defects: The effect on midwifery staff. Br J Midwifery. 2001;9(1):17-24.

91. Larsson EC, Fried S, Essén B, Klingberg-Allvin M. Equitable abortion care – A challenge for health care providers. Experiences from abortion care encounters with immigrant women in Stockholm, Sweden. Sex Reprod Healthc. 2016;10:14-8.

92. Mizuno M. Confusion and ethical issues surrounding the role of Japanese midwives in childbirth and abortion: A qualitative study. Nurs Health Sci. 2011;13(4):502-6.

93. Puri MC, Raifman S, Khanal B, Maharjan DC, Foster DG. Providers' perspectives on denial of abortion care in Nepal: A cross sectional study. Reprod Health. 2018;15(1):170.

94. Zaręba K, Banasiewicz J, Rozenek H, Ciebiera M, Jakiel G. Emotional complications in midwives participating in pregnancy termination procedure: Polish experience. Int J Environ Res Public Health. 2020;17(8).

95. Halldén BM, Lundgren I, Christensson K. Ten Swedish midwives' lived experiences of the care of teenagers' early induced abortions. Health Care Women Int. 2011;32(5):420-40.

96. Lipp A. A woman centred service in termination of pregnancy: A grounded theory study. Contemp Nurse. 2008;31(1):9-19.

97. Perrin E, Berthoud M, Pott M, Vera AGT, Bianchi-Demicheli F. Views of healthcare professionals dealing with legal termination of pregnancy up to 12 WA in French-speaking Switzerland. Swiss Med Wkly. 2012;142:w13584.

98. Reeves JA, Goedken P, Hall KS, Lee SC, Cwiak CA. Anesthesia providers' perspectives on abortion provision: Deductive findings from a qualitative study. Int J Obstet Anesth. 2022;49:103239.

99. Graham RH, Mason K, Rankin J, Robson SC. The role of feticide in the context of late termination of pregnancy: A qualitative study of health professionals' and parents' views. Prenat Diagn. 2009;29(9):875-81.

100. Purcell C, Cameron S, Lawton J, Glasier A, Harden J. Contraceptive care at the time of medical abortion: Experiences of women and health professionals in a hospital or community sexual and reproductive health context. Contraception. 2016;93(2):170-7.

101. Oelhafen S, Monteverde S, Cignacco E. Exploring moral problems and moral competences in midwifery: A qualitative study. Nurs Ethics. 2019;26(5):1373-86.

102. Lamichhane P, Harken T, Puri M, Darney PD, Blum M, Harper CC, et al. Sex-selective abortion in Nepal: A qualitative study of health workers' perspectives. WHI. 2011;21(3):S37-S41.

103. Lipp A. Self-preservation in abortion care: A grounded theory study. J Clin Nurs. 2011;20(5-6):892-900.

104. Lipp A. Conceding and concealing judgement in termination of pregnancy: A grounded theory study. J Res Nurs. 2010;15(4):365-78.

105. Mizuno M, Kinefuchi E, Kimura R, Tsuda A. Professional quality of life of Japanese nurses/midwives providing abortion/childbirth care. Nurs Ethics. 2013;20(5):539-50.
